# Normal radial migration and lamination are maintained in dyslexia-susceptibility candidate gene homolog *Kiaa0319* knockout mice

**DOI:** 10.1007/s00429-016-1282-1

**Published:** 2016-08-10

**Authors:** Isabel Martinez-Garay, Luiz G. Guidi, Zoe G. Holloway, Melissa A. G. Bailey, Daniel Lyngholm, Tomasz Schneider, Timothy Donnison, Simon J. B. Butt, Anthony P. Monaco, Zoltán Molnár, Antonio Velayos-Baeza

**Affiliations:** 1 Department of Physiology, Anatomy, and Genetics, University of Oxford, Oxford, OX1 3QX UK; 2 Division of Neuroscience, School of Biosciences, Cardiff University, Cardiff, UK; 3 Wellcome Trust Centre for Human Genetics, University of Oxford, Oxford, OX3 7BN UK; 4 Strathclyde Institute of Pharmacy and Biomedical Sciences, University of Strathclyde, Glasgow, UK; 5 School of Pharmacy and Biomedical Sciences, University of Central Lancashire, Preston, UK; 6 Office of the President, Ballou Hall, Tufts University, Medford, MA 02155 USA

**Keywords:** *Kiaa0319*, Dyslexia, Neuronal migration, Anatomy, Cerebral cortex, Development

## Abstract

Developmental dyslexia is a common disorder with a strong genetic component, but the underlying molecular mechanisms are still unknown. Several candidate dyslexia-susceptibility genes, including *KIAA0319*, *DYX1C1,* and *DCDC2*, have been identified in humans. RNA interference experiments targeting these genes in rat embryos have shown impairments in neuronal migration, suggesting that defects in radial cortical migration could be involved in the disease mechanism of dyslexia. Here we present the first characterisation of a *Kiaa0319* knockout mouse line. Animals lacking KIAA0319 protein do not show anatomical abnormalities in any of the layered structures of the brain. Neurogenesis and radial migration of cortical projection neurons are not altered, and the intrinsic electrophysiological properties of *Kiaa0319*-deficient neurons do not differ from those of wild-type neurons. *Kiaa0319* overexpression in cortex delays radial migration, but does not affect final neuronal position. However, knockout animals show subtle differences suggesting possible alterations in anxiety-related behaviour and in sensorimotor gating. Our results do not reveal a migration disorder in the mouse model, adding to the body of evidence available for *Dcdc2* and *Dyx1c1* that, unlike in the rat in utero knockdown models, the dyslexia-susceptibility candidate mouse homolog genes do not play an evident role in neuronal migration. However, KIAA0319 protein expression seems to be restricted to the brain, not only in early developmental stages but also in adult mice, indicative of a role of this protein in brain function. The constitutive and conditional knockout lines reported here will be useful tools for further functional analyses of *Kiaa0319*.

**Electronic supplementary material:**

The online version of this article (doi:10.1007/s00429-016-1282-1) contains supplementary material, which is available to authorized users.

## Introduction

Developmental dyslexia or reading disability (RD) is a neurodevelopmental disorder, defined by difficulty in accurate or fluent word recognition and spelling despite adequate instruction and intact sensory abilities. It is estimated to affect around 5–17 % of the population (Pennington and Bishop 2009; Peterson and Pennington 2012), and it is likely the result of multiple genetic and environmental interactions (Paracchini et al. 2007). Several candidate dyslexia susceptibility genes have been identified through linkage analysis and association studies, including *DYX1C1, DCDC2,* and *KIAA0319* (reviewed in (Carrion-Castillo et al. 2013)). Amongst them, *KIAA0319* is a particularly strong candidate, supported by independent association studies (Francks et al. 2004; Cope et al. 2005; Harold et al. 2006; Luciano et al. 2007; Paracchini et al. 2008; Couto et al. 2010; Scerri et al. 2011; Venkatesh et al. 2013), and functional evidence of a 40–50 % reduction in gene expression in the presence of the minor allele of a specific variant, rs9461045, which was shown to introduce a binding site for the transcription factor OCT1 in the *KIAA0319* promoter (Paracchini et al. 2006; Dennis et al. 2009). In addition, recent findings suggest genetic association of *KIAA0319* with other neurodevelopmental disorders (e.g. specific language impairment, verbal apraxia, and dyscalculia) (Newbury et al. 2011; Worthey et al. 2013; Mascheretti et al. 2014).

The underlying causes of RD are still a matter of debate. Deficits in neuronal migration were suggested over 30 years ago when cortical anomalies were found in patients with dyslexia in postmortem histopathological studies (Galaburda and Kemper 1979; Galaburda et al. 1985). Cortical projection neurons generated in the ventricular zone migrate radially to form the cortical plate during development (Rakic 1972, 1978), and this migration process is critical for the correct lamination and function of the cerebral cortex. Once the first dyslexia susceptibility candidate genes were identified, these early findings prompted the analysis of their roles during cortical development, particularly in migration. The studies were carried out in rats, using in utero electroporation of small hairpin RNAs (shRNAs) to downregulate gene expression. For *Kiaa0319*, these treatments led to migration defects (Paracchini et al. 2006; Peschansky et al. 2010; Adler et al. 2013) with many targeted neurons failing to migrate and giving rise to white matter heterotopias in postnatal animals, as well as hippocampal dysplasia in some cases. In addition, behavioural studies performed with these shRNA-treated rats have also found deficits in spatial learning and rapid auditory processing, particularly with speech-like sounds (Szalkowski et al. 2012; Centanni et al. 2014a, b). Similar results regarding migration impairment, obtained following the same experimental approach with other candidate genes such as *DCDC2* and *DYX1C1,* led to the hypothesis that deficits in neuronal migration during development predispose to RD (reviewed in (Gabel et al. 2010)). The results obtained in rats after gene expression knockdown suggested that alteration of neuronal migration and/or cortical lamination would be expected after disruption of the homologous genes in the mouse. However, no anatomical evidence supporting this hypothesis has been found in *Dcdc2*- or *Dyx1c1*-deficient mice (Wang et al. 2011; Rendall et al. 2015). Interestingly, electrophysiological and behavioural changes have been reported in both cases (Gabel et al. 2011; Che et al. 2014; Truong et al. 2014; Rendall et al. 2015; Che et al. 2015) indicative of a role for these proteins in processes other than neuronal migration.

The main product of the *KIAA0319* gene is a transmembrane protein that can localise to the plasma membrane (Velayos-Baeza et al. 2008). It follows the clathrin-mediated endocytic route (Levecque et al. 2009) and undergoes ectodomain shedding and intramembrane cleavage (Velayos-Baeza et al. 2010) generating fragments that can be released both intra- and extracellularly and that may act in signalling pathways. The KIAA0319 protein contains two cysteine-rich regions and 5 polycystic kidney disease (PKD) motifs in its extracellular domain (Velayos-Baeza et al. 2007). The PKD domains have been implicated in homophilic cell–cell adhesion (Ibraghimov-Beskrovnaya et al. 2000), suggesting that KIAA0319 could be involved in adhesion itself. *KIAA0319* is expressed in the developing and adult brain both in humans and in rodents (Paracchini et al. 2006; Velayos-Baeza et al. 2007; Peschansky et al. 2010). This expression pattern and the potential role in adhesion are compatible with an involvement of KIAA0319 in neuronal migration, as suggested by the shRNA experiments performed in rats (Paracchini et al. 2006).

Here, we report the generation and initial characterisation of a *Kiaa0319* knockout mouse line. Our results show that *Kiaa0319* knockout mice do not display any overt anatomical, neurological or behavioural deficits, and suggest that either KIAA0319 is not a major factor influencing mouse corticogenesis or the other proteins could potentially compensate for its absence. These results may reflect species-specific differences and do not necessarily contradict those obtained in rats nor have to be a reflection of what would happen in primates/humans if *KIAA0319* gene was disrupted in a similar way.

## Materials and methods

### Animals

**Fig. 1:**
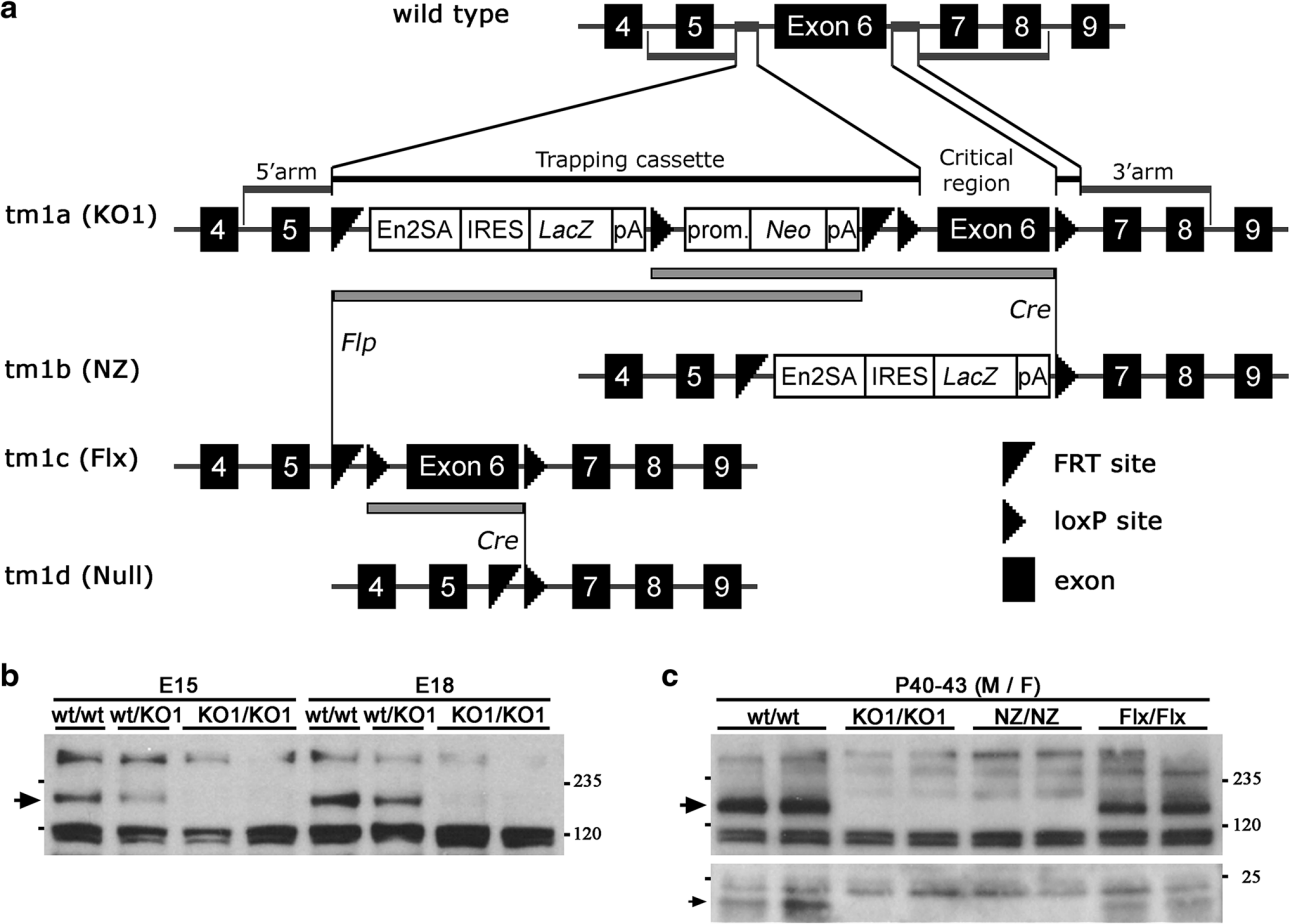
Generation and characterisation of *Kiaa0319*-targeted mice. **a** Schematic representation of strategy followed for targeting exon 6 of the *Kiaa0319* gene to obtain a “knockout first” (*tm1a* or KO1) allele. Alleles “Null-lacZ” (*tm1b* or NZ) and “floxed” (*tm1c* or Flx) are obtained after Cre and Flp recombination, respectively. Further Cre recombination from Flx allele generates a “Null” (*tm1d* or Null) allele where exon 6 is deleted. **b** Western blotting analysis of brain lysates from E15 and E18 embryos confirms the presence at these stages of the KIAA0319 protein (*arrow*) in wild type mice and its absence in homozygous KO1 animals. Non-specific bands are detected due to cross-reactivity of R7 antiserum. 30 µg total protein per lane. **c** Western blotting analysis with R7 antibody of brain lysates from 6-week old mice shows the presence of the KIAA0319 protein (*arrow*,* top panel*) in adult wild type and Flx mice and its absence in homozygous KO1 and NZ animals. The same pattern is detected for a < 25 kDa band (*arrow*,* bottom panel*), probably corresponding to a C-terminal cleavage fragment. 50 µg total protein per lane. *M* male, *F* female

Mouse embryonic stem cells targeted at the *KIAA0319*-homologous mouse gene *D130043K22Rik* with a “knockout-first” (KO1) (Skarnes et al. 2011) allele (C57BL/6N-*D130043K22Rik* ^*tm1a(KOMP)Wtsi*^) were purchased from the Knock-Out Mouse Project (KOMP) repository at UC Davis, California (www.komp.org) and used to obtain male chimeras after blastocyst injections. Mouse lines carrying different alleles (*tm1a, tm1b, tm1c,* and *tm1d*), denoted here by *Kiaa0319*-*KO1*, -*NZ*, -*Flx,* and -*Null*, respectively, were generated as described in Supplementary Information and Fig. 1a. Unless specified otherwise, *Kiaa0319*-*NZ* mice were used as the “ko” or “mutant” line.

Animals were housed with constant access to water and standard food under a 12-h light/dark cycle in controlled constant temperature and humidity. The maintenance and testing of these animals were performed under the UK Home Office guidelines for the treatment of animals under scientific procedures and the local ethical review board at the University of Oxford.

### PCR, genotyping, and sequencing

Mouse genomic DNA was extracted from ear notches or small pieces of tail (from embryo and young pups culled for samples) as previously described (French et al. 2007) with minor modifications. In brief, one or two notches or tail pieces were incubated at 56 °C for 1–3 h in 0.1 ml lysis buffer (50 mM Tris–HCl pH = 8–8.5, 1 mM EDTA, 0.5 % Tween 20, 0.5 mg/ml Proteinase K), followed by 12-to-15-min incubation at 100 °C for Proteinase K inactivation, and 10-min centrifugation at 14,000 rpm. DNA was diluted 1:5 and arranged in 96-well plate format for genotyping using 5 µl of DNA and 20 µl of PCR mix [1× buffer, 2 mM MgCl_2_, 0.4 mM dNTPs, 0.4 µM primers, 1 % BioTaq DNA polymerase (Bioline Reagents)]. Long-range PCR for amplification of KO1 allele fragments was performed using SequalPrep Long polymerase (Life Technologies) following manufacturer’s instructions. Primers used for sequencing and genotyping are described in Table S1. The different primer combinations used for sequencing and/or genotyping are described in Figs. S2 and S4.

### Western blotting

Dissected tissue was disrupted into cold RIPA buffer without detergents (50 mM Tris–HCl pH = 7.5, 150 mM NaCl), containing 4× Complete EDTA-free protease inhibitor cocktail (Roche) with a dounce homogenizer, transferred onto an Eppendorf tube, mixed with an equal volume of RIPA buffer containing 2× detergents (50 mM Tris–HCl pH = 7.5, 150 mM NaCl, 2 % NP40, 1 % Na-deoxycholate, 0.2 % SDS) and incubated on ice for at least 1 h. After centrifugation at 6000×*g* for 10 min at 4 °C, supernatant was collected and saved at −80 °C until used for Western blotting as previously described (Velayos-Baeza et al. 2008). Specific antiserum R7 (Velayos-Baeza et al. 2010) was used for detection of mouse KIAA0319 protein.

### Expression constructs

Amplification and cloning of the full-length mouse *Kiaa0319* cDNA have been previously described (Velayos-Baeza et al. 2007). The insert of plasmid pcDmKAm, obtained after sub-cloning into pcDNA4-TOmycHisA vector (Life Technologies), was amplified with primers mKiaa-F-XhoI and mKiaa-R-EcoRI (Table S1) and sub-cloned into pCIG (Megason and McMahon 2002) to generate pCIG-mKiaa0319. Cre recombinase was amplified from pML78 (which contains the ßactin-Cre construct described in (Lewandoski et al. 1997)) with primers Cre-F-XhoI and Cre-R-EcoRI (Table S1) and cloned into pCIG to generate pCIG-Cre. All inserts generated by PCR were verified by sequencing of the final construct.

### Histology and immunostaining

Embryonic brains were dissected and fixed in 4 % paraformaldehyde (PFA) overnight (O/N) at 4 °C. Postnatal day 2 pups (P2) and P10 pups were anesthetised with pentobarbitone and perfused with 5 or 10 ml of PBS followed by 5 or 10 ml of 4 % PFA, respectively, before brain dissection and O/N post-fixation in 4 % PFA. Brains were sectioned coronally at 50 μm or sagitally at 100 μm with a vibrating microtome (VT1000S; Leica). Immunohistochemistry was performed on a minimum of 3 floating sections per brain. For immunostaining, sections were incubated with blocking buffer (4 % BSA, 3 % Normal Goat Serum, 0.1 % Triton-X-100 in PBS) for a minimum of 60 min at room temperature (RT). Primary antibody incubation was performed in blocking buffer O/N at 4 °C after which sections were washed three times with PBS. Secondary antibodies (Alexa Fluor^®^ conjugated, Molecular Probes) were added in blocking buffer (1/500) for 1–2 h at RT. Sections were washed three times with PBS and mounted with ProLong Gold mounting medium (Molecular Probes). Nuclei were counterstained with DAPI (Molecular Probes). Primary antibodies used for immunostaining are listed in Table S2. Images were captured using a confocal laser-scanning microscope (LSM710, Zeiss or Leica TCP SP8). For quantification, 100-μm wide equivalent coronal sectors were selected, cortical thickness measured and divided into ten equally sized bins or in relevant subdivisions.

### In utero electroporation

Timed pregnant mice were anesthetised with isoflurane and their uterine horns exposed. Plasmid DNA (2 µg/µl) was injected into the embryos’ lateral ventricles. For electroporation, 5 pulses of 50–70 ms separated by 950 ms were applied at 35 V for E13.5 embryos and at 40 V for E14.5 embryos. Embryos were allowed to develop in utero for the indicated time. For analysis, embryonic brains were fixed in 4 % PFA O/N at 4 °C. For postnatal analysis, pups were anesthetised with pentobarbitone and fixed by transcardial perfusion with 10 ml PBS followed by 10 ml 4 % PFA before dissection and post-fixation. Brains were sectioned coronally at 100 μm with a vibrating microtome (VT1000S; Leica). At least four animals from three separate experiments were analysed for each condition.

### Acute in vitro slice recordings

Slices were prepared from 12-to-16-day-old *Kiaa0319* ^+/+^, *Kiaa0319* ^*F*/+^, and *Kiaa0319* ^*F/F*^ mice that had been co-electroporated in utero at E13.5 with pCIG-Cre and pCAG-RFP. Mice were deeply anesthetised with 4 % isoflurane (in 100 % O_2_) before decapitation and dissection of the brain in ice-cold, normal artificial CSF (ACSF) (125 mM NaCl, 2.5 mM KCl, 25 mM NaHCO_3_, 1.25 mM NaH_2_PO_4_, 1 mM MgCl_2_, 2 mM CaCl_2_, 20 mM Glucose; pH equilibrated with 95 % O_2_/5 % CO_2_]. Coronal slices (350 µm thick) were cut in ice-cold ACSF using a vibratome (Vibratome 3000 Plus; The Vibratome Company) before being individually transferred to an incubation chamber containing normal ACSF maintained at RT, where they were stored for a minimum of 1 h before recording.

### Whole-cell patch-clamp electrophysiology

Whole-cell tight-seal patch-clamp recordings were made in normal ACSF at RT from randomly selected RFP-positive infragranular pyramidal cells (>50 µm deep) in somatosensory cortices (S1 and S2). Patch electrodes were made from borosilicate glass (5–8 MΩ; Harvard Apparatus) and filled with a solution containing 128 mM K-gluconate, 4 mM NaCl, 0.3 mM Li-GTP, 5 mM Mg-ATP, 100 nM CaCl_2_,10 mM HEPES, and 1 mM glucose. Standard electrophysiological protocols were followed throughout. The intrinsic electrophysiological properties of the recorded cells were ascertained using the current clamp configuration using injection of 500-ms duration depolarising and hyperpolarising current steps (0.1–0.2 Hz) (MultiClamp 700B; Molecular Devices) and analysed offline (Clampfit v10). All parameters were measured on at least three occasions for each cell.

### Behavioural testing

A cohort of adult (22–28 weeks old) male and female homozygous, heterozygous, and wild-type littermates from *Kiaa0319*-*NZ* heterozygous breeding was used. Mice were housed 3–6 per cage. All experiments were conducted between 08:00 and 16:00 h, always during the light phase.

Groups of 6–9 mice per genotype per sex were used (Table S3). Behavioural testing included a series of consecutive tests separated by several days without testing: elevated plus maze, open field, locomotor habituation, rotarod, inverted screen, grip strength/weight lifting test, spontaneous alternations, spatial novelty, object recognition, light/dark box, sociability and preference for social memory, and auditory sensorimotor gating. Experimental procedures were based on previously published protocols (Schneider et al. 2012; Ufartes et al. 2013); a more detailed description for each of the tests can be found in the Supplementary Information.

## Results

### Generation of *Kiaa0319*-targeted mice

To study the putative role in cortical development postulated for human and rat *KIAA0319* genes, we generated mice targeted at the homologous *D130043K22Rik locus*. All animals used in this work were derived from a single male chimera. Although the original ES clones had passed KOMP quality control tests, we analysed the *Kiaa0319*-*KO1* allele in the obtained mice to confirm all important elements were as expected. This was carried out by amplification of four overlapping fragments (KO1-1 to KO1-4), specific to the *Kiaa0319*-*KO1* allele, covering the whole region included in the targeting vector plus short flanking regions to both homology arms (Figs. S2a, S2b, S3, S4). PCR amplification of fragments with combinations of a KO1-allele specific primer and a flanking sequence specific primer (PCRs KO1-1 and KO1-4) confirmed the correct targeting into the *Kiaa0319 locus*. After sequencing, only minor discrepancies were found (Fig. S3).

Homozygous *Kiaa0319*-*KO1* (*tm1a* allele) and *Kiaa0319*-*NZ* (*tm1b* allele) mice are viable, show no obvious differences with heterozygous and wild-type littermates, and breed normally. Western blotting analysis of brain lysates from E15 and E18 embryos showed that the specific band corresponding to the KIAA0319 protein was absent in homozygous samples (Fig. 1b). We additionally obtained *Kiaa0319*-*Flx* mice (*tm1c* allele), with conditional KO potential and in which the KIAA0319 protein could now be detected (Fig. 1c), and *Kiaa0319*-*Null* mice (*tm1d* allele) in which exon 6 is removed. As with *KO1* and *NZ* mice, *Kiaa0319*-*Null* mice do not show any obvious phenotype and do not have detectable KIAA0319 protein (Fig. S6c).

Western blotting analysis of lysates from different mouse tissues shows that the KIAA0319 protein is not only detected at early developmental stages (Fig. 1b) but also in adult brain (Fig. 1c). Interestingly, the same pattern of the presence/absence of signal found for the ~170 kDa band, corresponding to the glycosylated full-length protein, can be detected for a small, under 25-kDa band (Fig. 1c, bottom panel) probably corresponding to a C-terminal cleavage fragment (Velayos-Baeza et al. 2010). Specific KIAA0319 signal was not detected in other tissues (liver, kidney, spleen, lung, and heart) except perhaps weekly in testis (Fig. S2d), suggesting that the function of this protein is probably restricted to the nervous system.

We selected *Kiaa0319*-*NZ* mice as the ko line for characterisation, and used *Kiaa0319*-*Flx* mice to occasionally knockout the *Kiaa0319* gene at specific locations.

### Neurogenesis is not affected in *Kiaa0319* ko animals

**Fig. 2:**
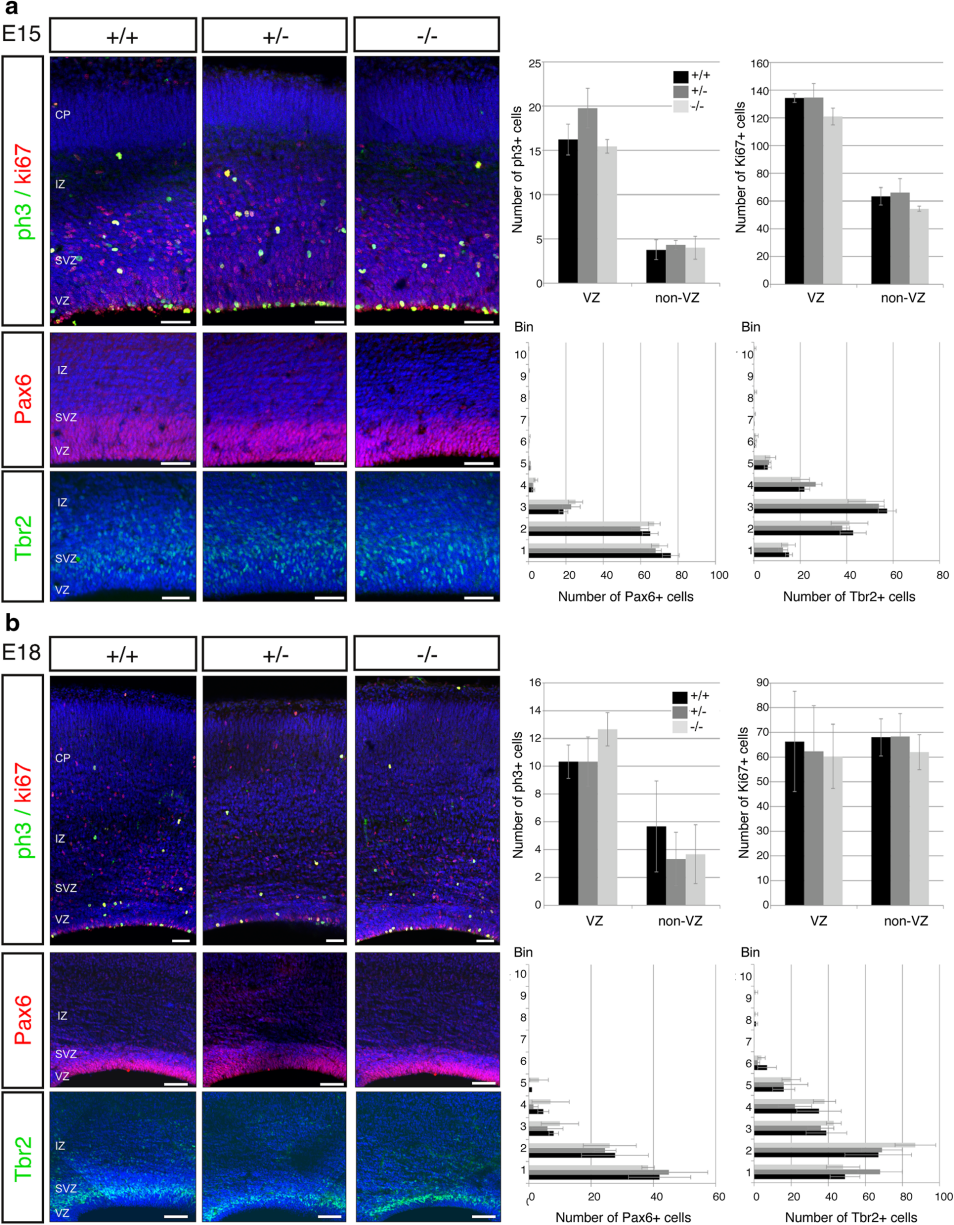
Neurogenesis is not altered in *Kiaa0319* mutants. **a** Immunostaining of *Kiaa0319* +/+, +/− and −/− embryos at E15.5. There are no differences between the three genotypes in the numbers or distribution of dividing (pH3 + , *green*) or cycling (Ki67 + , *red*) cells along the cortical wall (*upper panels*). Staining for Pax6 (*red*,* middle panels*) and Tbr2 (*green*,* lower panels*) also shows no changes in *Kiaa0319* deficient animals. Nuclei were stained with DAPI (*blue*). Quantifications of number of cells per 100-μm wide sections in the ventricular zone and rest of cortical wall (upper panels) or per bin (*middle* and* lower panels*) are shown on the right. **b** Immunostaining of +/+, +/− and −/− embryos at E18.5. The number of dividing (pH3 + , *green*) or cycling (Ki67 + , *red*) cells and their distribution, quantified as number of cells per 100-μm wide section in the ventricular zone and the rest of the cortical wall, shows no variation between +/+, +/− and −/− animals (*upper panels*). Immunostaining against Pax6 (*red*,* middle panels*) and Tbr2 (*green*,* lower panels*) shows similar numbers and distribution of positive cells in all three conditions. Nuclei were stained with DAPI (*blue*). Quantifications are shown on the *right*. *VZ* ventricular zone, *SVZ* subventricular zone, *IZ* intermediate zone, *CP* cortical plate. *Scale bars* 75 μm

*Kiaa0319* has been reported to be expressed in the VZ of the developing cortex both in mice and humans by RNA in situ hybridization experiments (Paracchini et al. 2006; Peschansky et al. 2010). This expression pattern in progenitor cells suggests that *Kiaa0319* might play a role during neurogenesis. To investigate this possibility, we analysed embryonic brains of wild-type, heterozygous, and homozygous *Kiaa0319* ko animals at E15.5 and E18.5. Brain sections were stained for markers Ki67 and pH3 to label cells in the cell cycle and in mitosis, respectively (Fig. 2). Quantification of the number of positive cells for these two markers revealed no significant differences between the three genotypes (Fig. 2). To check whether the proportion of RG cells and intermediate progenitors, the two main progenitor types in mice, was altered in *Kiaa0319* mutant animals, we stained E15.5 and E18.5 sections with antibodies against Pax6 and Tbr2, respectively. Again, quantification of the Pax6- or Tbr2-positive cells did not show any significant differences between genotypes (Fig. 2). In addition, the thickness of the cortical wall in E18.5 brain slices did not differ between the three conditions (data not shown). Together, these results suggest that *Kiaa0319* is not playing a major role in neurogenesis during cortical development or that, alternatively, its absence may be functionally compensated by other proteins.

### Normal lamination of cortex, hippocampus and cerebellum in *Kiaa0319* ko mice

**Fig. 3:**
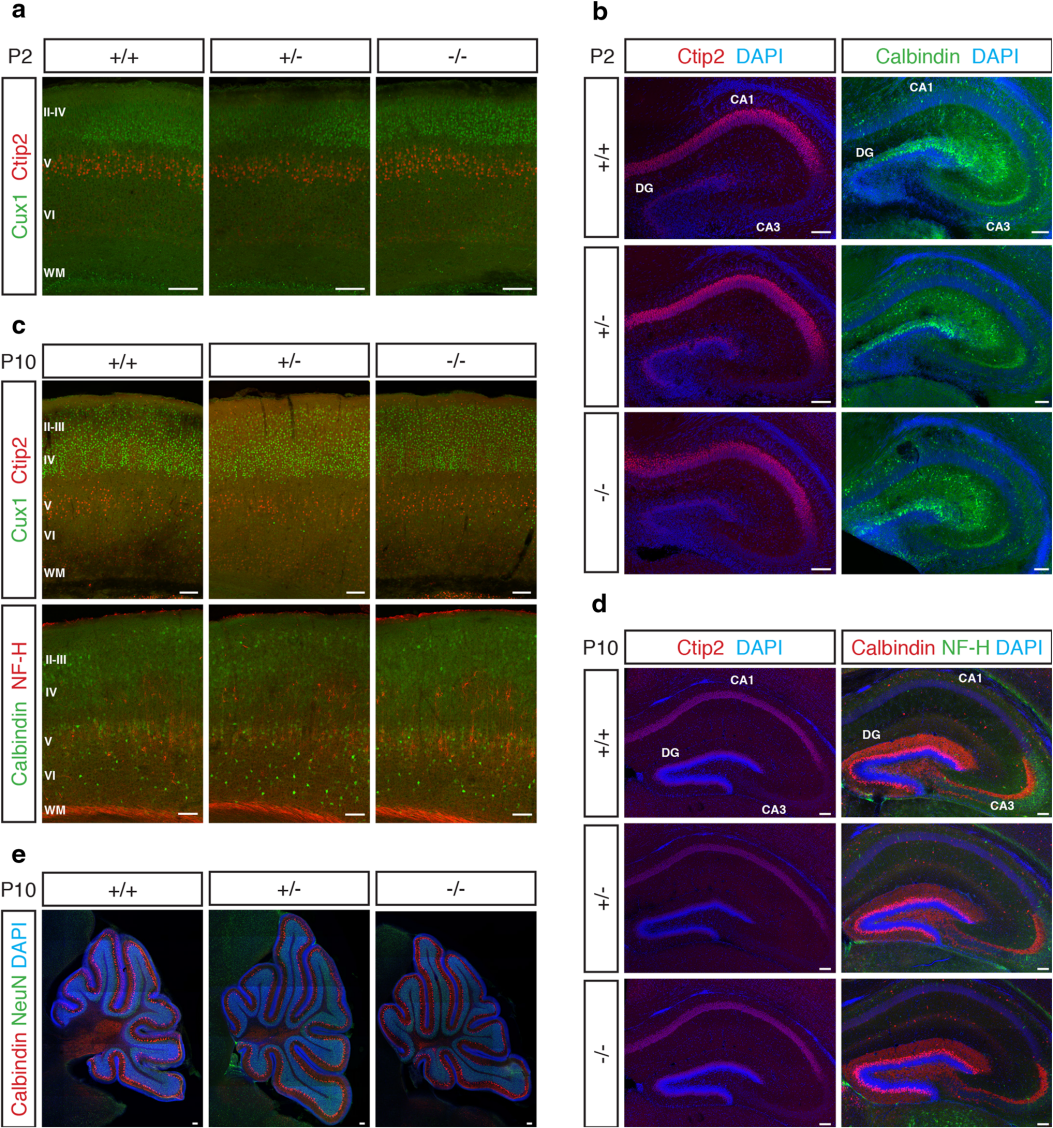
Normal lamination in layered brain regions of *Kiaa0319*-deficient mice. **a** Immunostaining with anti-Ctip2 (*red*) and anti-Cux1 (*green*) antibodies in the P2 somatosensory cortex of *Kiaa0319* +/+, +/− and −/− animals, revealing normal lamination of deep and upper layers. **b** P2 hippocampi stained for Ctip2 (*red*,* left panels*) and Calbindin (*green*,* right panels*), showing no differences between the three *Kiaa0319* genotypes. Nuclei were stained with DAPI (*blue*). **c** Upper panels: immunostaining against Ctip2 (*red*) and Cux1 (*green*) in P10 somatosensory cortex shows normal lamination in *Kiaa0319* deficient mice.* Lower panels* further staining against NF-H (*red*) and Calbindin (*green*) confirms normal neuronal distribution in the mutants. **d** No lamination defects are apparent in P10 hippocampi stained for Ctip2 (*red*, left panels) or Calbindin (*red*) and NF-H (*green*) (*right panels*). Nuclei were stained with DAPI (*blue*). **e** Immunostaining against NeuN (*green*) and Calbindin (*red*) shows normal foliation and lamination of P10 cerebella in +/+, +/− and −/− animals. Nuclei were stained with DAPI (*blue*). Cortical layers are labelled with *roman numbers* (II to VI); *WM* *white* matter, *DG* dentate gyrus, *CA1*–*CA3* Cornu Ammonis areas 1–3. *Scale bars* 100 μm

Functional experiments using in utero electroporation of shRNA against *Kiaa0319* in rat embryos have shown a defect in radial migration following *Kiaa0319* knockdown: groups of electroporated neurons fail to migrate and form heterotopias in the white matter (Paracchini et al. 2006; Peschansky et al. 2010; Szalkowski et al. 2012; Adler et al. 2013). These experiments suggest that *Kiaa0319* plays an important role in the migration of newborn neurons into the CP, which could impact on the correct formation of the cortical layers. However, because in utero electroporation only targets a small percentage of neurons, effects on cortical lamination could not be analysed by this method. To check if a partial or total absence of *Kiaa0319* affects the final position of projection neurons, we assessed cortical lamination at P2, when deep layer neurons have finished migrating but upper layer neurons are still on their way, and at P10, when all projections neurons have reached their final position. Staining of brain sections with antibodies against Ctip2 and Cux1, to label layer V neurons and upper layer neurons, respectively, showed no aberrant distribution of cells in the *Kiaa0319* heterozygous or homozygous ko animals. Ctip2 positive cells were located in a band below Cux1 positive cells that occupied the upper part of the cortical plate at P2 (Fig. 3a). A similar distribution could be seen at P10, where layer IV could also be readily identified by the presence of the barrel cortex in all three conditions (Fig. 3c). Further staining for NF-H at P10 also showed no differences in the location and shape of labelled neurons and Calbindin staining revealed similar interneuron distribution across the three genotypes (Fig. 3c). To analyse whether other layered structures could be affected by a lack of KIAA0319 protein, we performed immunohistochemistry with anti-Ctip2 and anti-Calbindin antibodies to stain CA1 and the Dentate Gyrus of the hippocampus at P2 and P10 (Fig. 3b,d). Again, no migration defects were apparent, and no differences in the appearance or distribution of cells could be identified between wild-type, heterozygous, and homozygous *Kiaa0319* ko animals. Finally, we looked at the cerebellum of P10 animals, using antibodies against Calbindin and NeuN (Fig. 3e). Calbindin-positive Purkinje cells were correctly positioned, and no lamination defects could be seen in the heterozygous or homozygous ko animals. To account for the possibility that any migration defects could be transitory and not visible at postnatal stages, we repeated the cortical stainings on sections of E15.5 (Ctip2) and E18.5 (Ctip2 and Cux1) brains, but the distribution of the labelled cells was the same in all three conditions (Fig. S5). Together, these results demonstrate that a partial or complete reduction in the levels of KIAA0319 protein in the mouse does not affect migration in any of the layered structures of the brain.

### Acute elimination of KIAA0319 protein by Cre recombination does not affect radial migration of projection neurons

**Fig. 4:**
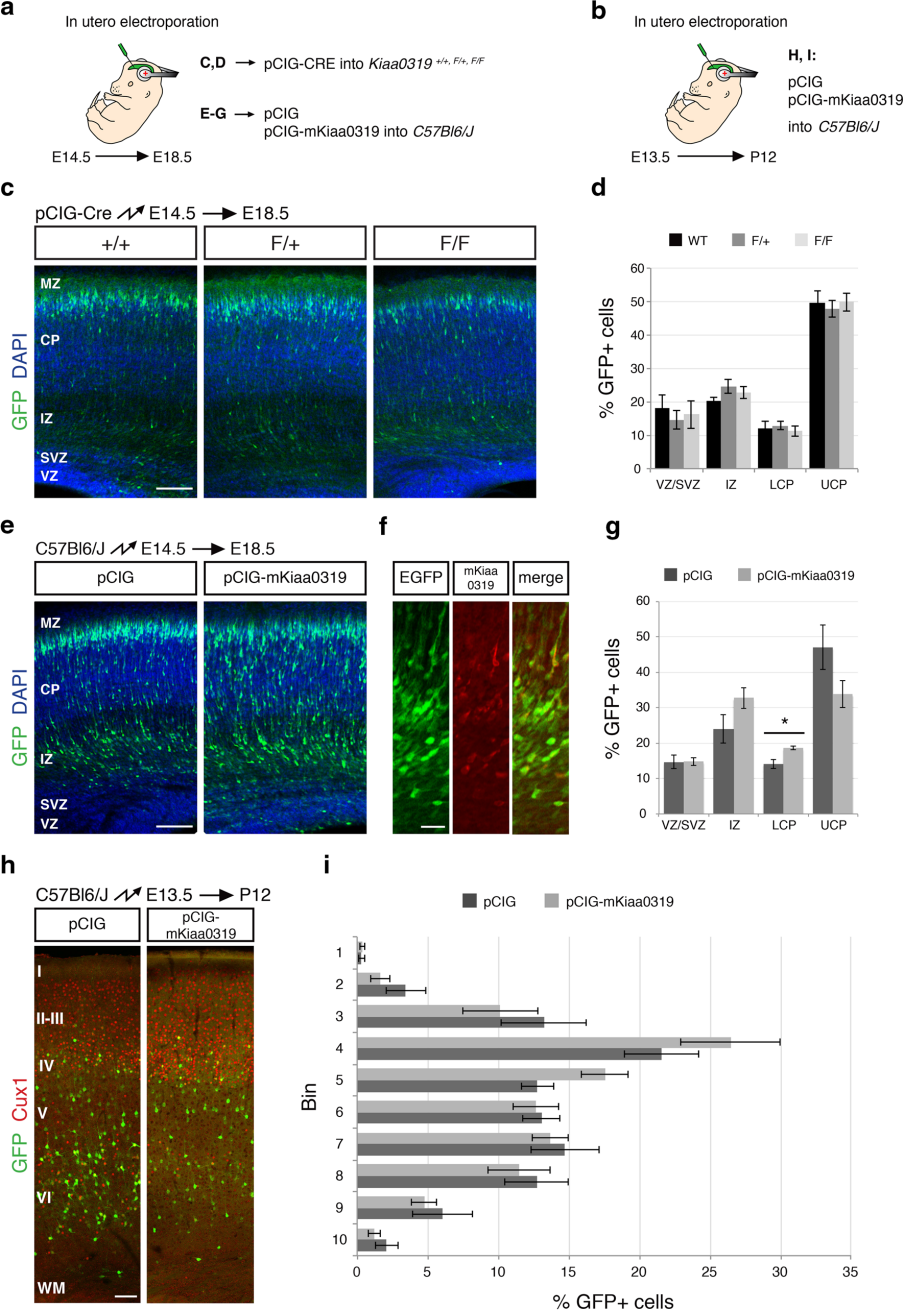
Effects on radial migration upon *Kiaa0319* depletion or overexpression. **a** Illustration of the strategy to study the effects of altered *Kiaa0319* levels on neuronal migration at embryonic stages. To reduce KIAA0319 levels, embryos from wild-type (+/+), heterozygous (F/+), and homozygous (F/F) floxed animals were electroporated in utero at E14.5 with pCIG-Cre. For overexpression, C57Bl/6J embryos were electroporated at E14.5 with either pCIG or pCIG-mKiaa0319. Neuronal position was analysed at E18.5. **b** Strategy to assess the effect of *Kiaa0319* overexpression on migration at postnatal stages. C57Bl/6J embryos were electroporated at E13.5 with either pCIG or pCIG-mKiaa0319 and analysed at postnatal day 12. **c** Representative images of coronal sections of embryos electroporated with pCIG-Cre. Electroporated neurons are shown in *green* and nuclei (DAPI stained) in *blue*. KIAA0319 depletion at E14.5 does not alter the radial migration of cortical projection neurons. **d** Quantification of the percentage of electroporated neurons in the VZ/SVZ, IZ, LCP, and UCP shown in **c**. Neurons were counted in three slices of 7 (F/F), 8 (F/+) or 5 (+/+) electroporated brains originating from three separate experiments. The data represent mean ± SEM. No statistically significant differences were found by one-way ANOVA analysis. **e** Representative images of coronal sections of embryos electroporated with pCIG or pCIG-mKiaa0319. Electroporated neurons are shown in *green* and nuclei (DAPI stained) in *blue*. *Kiaa0319* overexpression slightly impairs migration. **f** Immunohistochemistry to detect the overexpressed KIAA0319 protein. Neurons electroporated with pCIG-mKiaa0319 express EGFP (*green*) and stain with anti-KIAA0319 R5 antibody (*red*). **g** Quantification of the percentage of electroporated neurons in the VZ/SVZ, IZ, LCP, and UCP shown in **e**. The data represent mean ± SEM. *p < 0.05 by Student’s *t* test. Three slices from five brains coming from three separate electroporations were counted for each condition. **h** Representative images of coronal sections of embryos electroporated at E13.5 with pCIG or pCIG-mKiaa0319 and analysed at P13. Electroporated neurons are shown in *green*. Cux1 staining to label upper cortical layers is shown in *red*. No difference in the distribution of targeted neurons is apparent. **i** Quantification of the distribution of electroporated neurons shown in **h**. The data represent mean ± SEM. No statistically significant differences were found for any of the bins by Student’s *t* test. Three slices from four brains coming from three separate electroporations were counted for each condition. *MZ* marginal zone, *LCP* lower half of the cortical plate, *UCP* upper half of the cortical plate, other abbreviations as in Figs. 2, 3. *Scale bars* 100 μm

The lack of lamination defects in the *Kiaa0319* ko mouse is in contradiction with the shRNA electroporation data obtained in rat by other laboratories (Paracchini et al. 2006; Peschansky et al. 2010; Szalkowski et al. 2012; Adler et al. 2013). One possible explanation for this discrepancy could be the timing of *Kiaa0319* knockdown. Because in the ko animals the protein is missing from the beginning, compensatory mechanisms might be operating to counteract its absence by the time neuronal migration takes place. An acute knockdown of the protein right before migration commences, as achieved by shRNA electroporation, might overcome this putative compensation. To acutely eliminate KIAA0319 in migrating neurons, we carried out in utero electroporation experiments to deliver Cre-recombinase encoding plasmids into *Kiaa0319*-*Flx* embryos (Fig. 4a). Plasmid pCIG-Cre, expressing Cre-recombinase and EGFP under the control of a general promoter (Chicken Beta Actin), was electroporated at E14.5 into *Kiaa0319* ^+/+^, *Kiaa0319* ^*F*/+^, and *Kiaa0319* ^*F/F*^ embryos, and brains were harvested four days later, at E18.5 (Fig. 4c). The number of neurons in four different regions of the developing cortex (VZ/SVZ, IZ, lower CP, and upper CP) was quantified (Fig. 4d), but no significant differences could be found between the three conditions. Consistent with these results, there were no misplaced neurons in the white matter of *Kiaa0319* ^*F*/+^ and *Kiaa0319* ^*F/F*^ postnatal animals electroporated with pCIG-Cre at E13.5 (data not shown). Cre expression from the pCIG-Cre plasmid was verified by immunohistochemistry, confirming the presence of Cre recombinase in the electroporated cells (Fig. S6a). The lack of a suitable antibody to exclusively detect the endogenous KIAA0319 protein precluded us from confirming that the protein was effectively absent following Cre electroporation in brain slices. However, we tested that the *Kiaa0319*-*Null* allele can indeed be obtained from *Kiaa0319*-*Flx* allele after Cre recombination in primary cortical cultures transfected with the same plasmid (Fig. S6b), and Western blotting analysis of protein lysates obtained from these same cultures shows a reduced KIAA0319 protein signal (Fig. S6c, left).

### *Kiaa0319* overexpression delays radial migration, but does not affect final neuronal position

Overexpression of *Kiaa0319* has been reported to alter the final location of migrating neurons in rats, where cells electroporated at E15/16 with an overexpression construct occupied lower positions than control cells in the cortical plate at P21 (Peschansky et al. 2010). To check if we could detect a similar effect in mice, we electroporated pCIG-mKiaa0319 into wild-type embryos at E14.5 (Fig. 4a) and analysed the brains 4 days later (Fig. 4e). We confirmed overexpression of KIAA0319 protein using immunohistochemistry (Fig. 4f) and quantified the percentage of electroporated cells in the different zones of the cortical wall (Fig. 4g). We could see a tendency of *Kiaa0319* overexpressing cells to lag behind control electroporated cells, with an increase in the percentage of targeted cells both in the IZ (24.04 % for pCIG vs 32.75 % for pCIG-mKiaa0319) and the LCP (14.14 % vs 18.6 %), and a concomitant reduction in the neurons that had reached the UCP (47.15 % vs 33.83 %). Due to the high variability in the distribution of the neurons, only the differences in the LCP were significant. To assess how this delay might affect the final position of excitatory projection neurons within the cortex, we electroporated pCIG-mKiaa0319 into E13.5 embryos and checked the position of the targeted cells in somatosensory cortex at P12 (Fig. 4b, h). Electroporation at E13.5 targets neurons from different layers, allowing us to check the effect of *Kiaa0319* overexpression in different neuronal populations. The distribution of *Kiaa0319* overexpressing neurons across the cortex, analysed as the percentage of targeted cells in each of 10 equal bins, did not differ significantly from the distribution of control electroporated cells (Fig. 4i). These results suggest that *Kiaa0319*-overexpressing neurons are delayed in their migration to the cortical plate, but manage to reach their final position within the cortex postnatally.

### No changes in the intrinsic electrophysiological properties of cortical neurons are detected upon partial or total elimination of KIAA0319

**Fig. 5:**
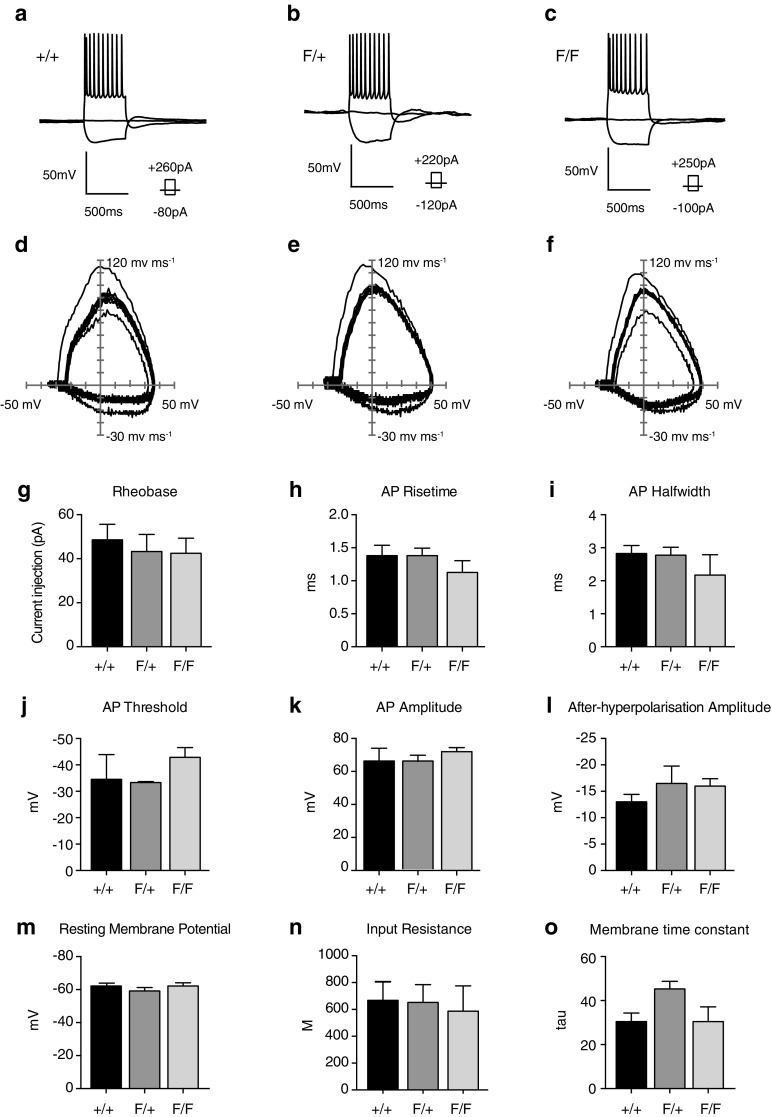
No changes in the intrinsic properties of cortical neurons upon partial or total elimination of *Kiaa0319*. **a–c** Superimposed responses of pyramidal cells to suprathreshold depolarising and hyperpolarising current injection for electroporated cells in wild-type (**a**; +/+), heterozygous (**b**; F/+) and homozygous (**c**; F/F) floxed animals. **d–f** Action potential phase (dV/dT) plots for 10 action potentials shown in panels **a–c**. **g–o** Active (**g–l**) and passive membrane properties (**m–o**) for electroporated cells recorded from +/+ (*n* = 6), F/+ (*n* = 5) and F/F (*n* = 5) animals. No significant differences were observed. The data represent mean ± SEM. Significance was assessed by Kruskal–Wallis test with Dunn’s correction for multiple comparisons

The absence of KIAA0319 does not seem to affect radial migration of cortical projection neurons, but it might impact their intrinsic electrophysiological properties. To lower the chances of having normal physiological maturation as a result of network compensation due to global knock out of *Kiaa0319,* we did not perform the analysis in ko animals. Instead, we electroporated pCIG-Cre, together with pCAG-RFP to enable the identification of electroporated neurons, into *Kiaa0319* ^+/+^, *Kiaa0319* ^*F*/+^, and *Kiaa0319* ^*F/F*^ embryos at E13.5 and harvested the electroporated animals between P12 and P16. Recordings were performed from acute in vitro brain slices. The response of electroporated cells to either depolarising or hyperpolarising current steps did not differ between genotypes (Fig. 5a–c). There was no significant difference in either action potential dynamics (Fig. 5d–l) or passive membrane properties (Fig. 5m–o). These results suggest that the basic intrinsic electrophysiological properties of the cohort of E13.5 cortical pyramidal neurons are unaltered following removal of KIAA0319.

### *Kiaa0319* mutants exhibit subtle behavioural anomalies in sensorimotor gating and anxiety measures

Despite the absence of overt anatomical or electrophysiological defects in *Kiaa0319* ko mice, underlying subtle circuit-level changes affecting their behaviour may exist. This hypothesis is supported by previous studies on mice mutated in other candidate dyslexia susceptibility homologous genes, such as *Dcdc2* and *Dyx1c1*. These animals do not show the expected migration deficits, and lack lamination defects or clear anomalies in the cortex; however, changes in learning, memory or auditory processing have been reported in both cases (Gabel et al. 2011; Truong et al. 2014; Rendall et al. 2015). Similarly, in utero knockdown of *Kiaa0319* in the embryonic rat cortex leads to spatial learning deficits and affect responses to complex acoustic stimuli (Szalkowski et al. 2012; Centanni et al. 2014a, b). We, therefore, decided to perform a general behavioural characterisation of the *Kiaa0319* mutant animals to elucidate functional effects of *Kiaa0319* knockout and conducted a standard series of mouse behavioural tests on a cohort of control and mutant mice.

**Fig. 6:**
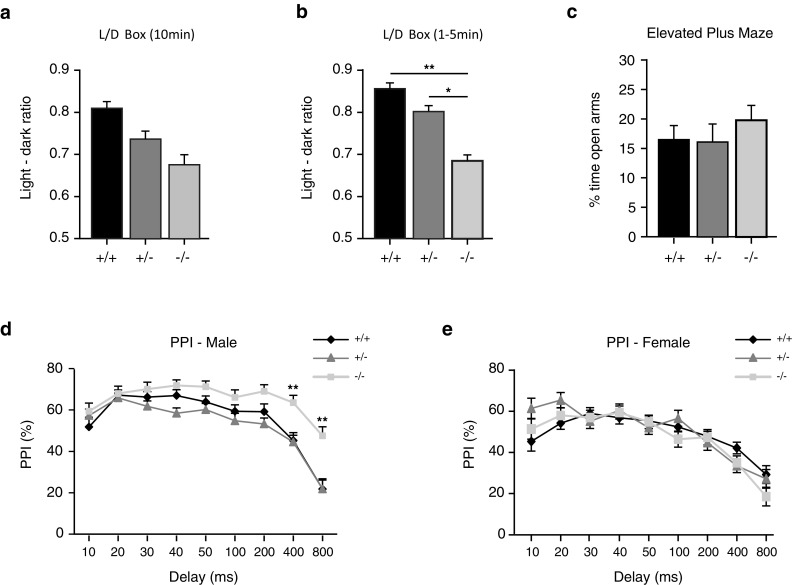
Subtle changes detected in light-induced anxiety and prepulse inhibition in *Kiaa0319* mutant mice. **a–c** Anxiety related behaviour measured in the *light/dark box* over the full 10 min duration of the test (**a**), in the initial 5 min of the *light/dark box* test (**b**) and in the elevated plus maze (EPM) (**c**) of *Kiaa0319* +/+ (*n* = 16; 17 for EPM), +/− (*n* = 17) and −/− (*n* = 14) mice. *Kiaa0319* −/− mice display reduced anxiety in the first 5 min of the *light/dark box* test when compared with +/+ and +/− animals. No significant difference in anxiety is observed between genotypes in the EPM. *Light*–*dark ratio* ratio of time spent in the dark *vs* time spent in the light. Post hoc statistically significant Bonferroni LSD results, *p ≤ 0.01, **p ≤ 0.001. **d–e** Prepulse inhibition/facilitation of the acoustic startle response in male (**d**) and female (**e**) *Kiaa0319* +/+ (male = 7, female = 7), +/− (male = 8, female = 4) and −/− (male = 7, female = 6) mice. Homozygous mutant (−/−) males show a deficit in prepulse facilitation at 400- and 800-ms gaps between prepulse and pulse compared with the other two genotypes. Post hoc statistically significant Bonferroni LSD results: homozygous (−/−) vs heterozygous (+/−) or wild-type (+/+), **p ≤ 0.001. The data represent mean ± SEM

There was no difference between genotypes in locomotor activity in the 60-min open field test (Fig. S7a), sociability and social novelty preference measured in the 3-chamber apparatus (Fig. S7b), locomotor habituation (Fig. S7c), or stress and motor coordination (inverted screen, weight lifting or accelerating rotarod) (Fig. S7d–f). There was also no difference in spatial and non-spatial learning and memory (spontaneous alternations using T maze, spatial novelty in the Y maze, and object recognition tests) (Fig. S7g–i); however, subtle differences were found in anxiety and sensorimotor gating (Fig. 6).

Anxiety was measured in the light/dark box and the elevated plus maze. In the light/dark box, significant interaction between genotype x sex x within-session period was found for the ratio of time spent in the dark *vs* light, a classic measure of anxiety, [*F*(2,417) = 2.969, *p* ≤ 0.05] (Fig. 6a). The difference was significant for the initial 5 min of the session [*F*(2,41) = 3.561, *p* ≤ 0.05] (Fig. 6b) with decreased anxiety in homozygous animals compared to both wild type (*p* < 0.001) and heterozygous animals (*p* < 0.01). Similarly, homozygous ko mice tended to spend more time in the open arms of the elevated plus maze, suggesting decreased anxiety, but this effect did not reach statistical significance (Fig. 6c).

We also assessed sensorimotor gating, i.e., an ability to filter out redundant stimuli, in the *Kiaa0319* mutant mice using the prepulse inhibition/facilitation (PPI/PPF) of the acoustic startle response. We used different latencies to the startle-eliciting stimulus (SES) to measure both prepulse facilitative and inhibitory effects on the reactivity to the SES. No differences in prepulse inhibition across genotypes and sex were detected, although there was a significant genotype x sex x stimulus type interaction [*F*(16,3423) = 1.92, *p* ≤ 0.05]. Further post hoc analysis revealed increased prepulse inhibition in homozygous males for stimuli with long gaps (400 and 800 ms) between prepulse and SES, compared both with wild-type and heterozygous mice (*p* ≤ 0.001) (Fig. 6d). There was no difference between females (Fig. 6e) or for any other stimuli combination in males.

Taken together, those data suggest the possibility of subtle alterations in anxiety-related behaviour and in sensorimotor gating resulting from *Kiaa0319*-deletion, although further analyses with larger cohorts and/or different tests may be necessary to replicate and extend these initial results.

## Discussion

*KIAA0319* is one of the strongest dyslexia susceptibility candidate genes identified so far, but its molecular and cellular functions are still unknown. Previous studies using shRNA against *Kiaa0319* in rat embryos have implicated this gene in the radial migration of cortical neurons during brain development (Paracchini et al. 2006; Peschansky et al. 2010; Adler et al. 2013). Here, we report the first characterisation of a *Kiaa0319* knockout mouse. Our analyses show no overt abnormalities in the anatomy, intrinsic electrophysiology or behaviour of mutant animals. Progenitor numbers and cycling cells are not altered during embryonic stages in *Kiaa0319*-deficient animals, and accordingly, there are no differences in cortical thickness or brain size. Migration is also not disrupted, and knockout animals show correct lamination of the cortex, hippocampus and cerebellum. Even an acute silencing of *Kiaa0319* by Cre electroporation into floxed animals does not disrupt radial migration of cortical projection neurons, which also show no differences in their intrinsic electrophysiological properties compared with wild-type cells. *Kiaa0319* overexpression in cortex delays radial migration, but does not affect final neuronal position. The behaviour of knockout and heterozygous animals in several tests, assessing cognitive learning and memory, auditory sensorimotor gating, social behaviour or anxiety, does not significantly differ from that of wild-type littermates.

Our data indicate that the function of *Kiaa0319* in mouse brain development is not as initially hypothesised, as it does not lead to gross defects in neuronal migration. The same conclusion can be drawn from the phenotypic data available from KOMP (www.kompphenotype.org), where the same mice have been obtained in parallel (in C57Bl/6N background), showing a lack of growth, gross histology or behavioural deficits, albeit with a relatively small sample size.

### Discrepancies between the outcomes of different migration experiments

Given the results of the shRNA experiments performed in rat embryos, we hypothesised that neurons lacking KIAA0319 would have their migratory behaviour affected. However, we have demonstrated this not to be the case, as no migration or lamination defects were detected neither in the constitutive knockout nor after Cre electroporation into floxed animals. A similar scenario was found for *Dcdc2* when the knockout mouse for this gene was first characterised (Wang et al. 2011), and for *Dyx1c1* when a forebrain-conditional knockout mouse was analysed (Rendall et al. 2015). Even though in utero electroporation in rat embryos of shRNAs against *Dcdc2* (Meng et al. 2005; Burbridge et al. 2008; Adler et al. 2013) or *Dyx1c1* (Wang et al. 2006; Threlkeld et al. 2007; Szalkowski et al. 2011, 2013) resulted in anatomical malformations, no migration deficits were found in *Dcdc2* knockout or *Emx1-Cre/Dyx1c1* ^*flox/flox*^ conditional knockout animals, nor in *Dcdc2* floxed mice following acute elimination of the protein by Cre electroporation. In this particular case, the authors reported increased developmental defects in radial migration and dendritic growth of layer III neurons in *Dcdc2* knockout animals upon RNA interference against Doublecortin (*Dcx*). Doublecortin family members, like *Dcx* and Doublecortin-like kinase 1 (*Dclk1*), play redundant roles in radial migration, as demonstrated by the migration defects of the *Dcx/Dclk1* double knockout when compared with the individual *Dcx* or *Dclk1* *null* animals, where cortical neurons migrate normally (Corbo et al. 2002; Deuel et al. 2006; Koizumi et al. 2006). The results obtained with *Dcdc2* knockouts could be explained by a similar mechanism, although a recent study has demonstrated that shRNAs used against doublecortin family members display significant off-target effects due to interference with endogenous microRNA processing in migrating neurons (Baek et al. 2014). The results of this study show that cortical migration is very sensitive to shRNA off-target effects and that even scrambled shRNAs can induce significant migration defects. Such off-target effects emerge from the way shRNAs are processed within cells, which lead to altered microRNA levels. These results could explain the discrepancy between our results and those previously published, although inter-species differences between mice and rats cannot be ruled out. In this context, it is also worth mentioning that the severe effects found in some human migration disorders, such as those caused by mutations in genes *LIS1*, *TUBA3,* or *DCX*, are not found in the corresponding KO models in mouse, in which no cortical migration defects, except for some minor hippocampal heterotopias, are detected (Corbo et al. 2002; Keays et al. 2007). Therefore, from our results, we cannot definitely conclude that alteration of the *KIAA0319* gene has no effect on neuronal migration in humans.

### Compensation hypothesis

Another possibility that needs to be considered is a potential compensation mechanism. It is worth noting that, at least in zebrafish, genetically altered animals that carry deleterious mutations can activate compensatory mechanisms that are not detected after transcriptional or translational knockdown (Rossi et al. 2015). In utero electroporation of Cre recombinase into homozygous, *Kiaa0319* floxed animals should circumvent this problem, but this approach also failed to generate a migration defect. *Kiaa0319* has one homologous gene, *AU040320*, which is expressed in adult mouse brain (Poon et al. 2011) and also during developmental stages (unpublished results). Its human orthologue, *KIAA0319L*, has also been associated with RD (Couto et al. 2008). Both mouse proteins are 45 % identical, 60 % similar, and could have redundant functions in tissues where both genes are expressed, including the brain. In utero electroporation of shRNA against *AU040320* in embryonic rats results in periventricular heterotopias in about 25 % of animals, suggesting that the *Kiaa0319* homologue could play a role in radial migration as well (Platt et al. 2013). We are currently exploring this possibility through the generation of *AU040320* single and *Kiaa0319/AU040320* double knockout mice.

### Electrophysiology

*Kiaa0319* encodes a transmembrane protein with a putative role in adhesion that is expressed during neuronal development. Even if it does not play a major role during neuronal migration, it is still likely to be involved in other processes, such as synaptogenesis or circuit formation. Our results indicate no changes in the intrinsic electrophysiological properties of KIAA0319-deficient cortical neurons, although our recordings were performed in cells depleted of KIAA0319 through in utero electroporation of Cre into floxed animals instead of using slices from knockout mice. This approach was chosen to prevent any potential compensatory mechanisms or other wider systemic effects.

Deletion of *Dcdc2*, another dyslexia susceptibility candidate gene, has recently been shown to affect cortical activity by altering the temporal firing pattern and functional connectivity in the neocortex (Che et al. 2014, 2015). Our results suggest that neurons depleted of KIAA0319 are following a normal intrinsic electrophysiological developmental trajectory, but more detailed studies on networks and synaptic changes would be required to assess a putative role of KIAA0319 in circuit formation and function.

### Behavioural analyses

At the behavioural level, our results with *Kiaa0319* ko mice show no impairment in locomotor activity, motor coordination, strength, social behaviour, or spatial and non-spatial learning and memory; however, we have identified a significant difference in the sensorimotor abilities of mutant males in the prepulse inhibition/facilitation test. Male *Kiaa0319* knockouts exhibit enhanced prepulse inhibition of startle responses at long intervals between prepulse and SES, which at this stage can be interpreted as either a deficit in filtering out redundant stimuli, as control animals show decreased impact of the prepulse on SES for those long delays, or as an enhanced ability to link stimuli even with those long delays, i.e., enhanced sensorimotor gating. Answering this question will require further studies.

Our experiments also revealed a decreased light-induced anxiety in *Kiaa0319* homozygous mutant mice in the first five minutes of the light/dark box test. This initial stage of the test is the most informative as with time animals habituate to the environment and their anxiety decreases. However, no other test indicated a significant role for *Kiaa0319* in anxiogenic pathways. Although, on average, homozygous mutant mice spent more time than control animals in the open arms of the EPM, an indication of lower anxiety, the differences were not significant. Therefore, reduction of anxiety in *Kiaa0319* mutant mice should be interpreted with caution.

Studies conducted with rats following in utero electroporation to knockdown *Kiaa0319* (Szalkowski et al. 2012; Centanni et al. 2014a, b) have reported spatial learning deficits and impaired auditory processing but normal working memory using behavioural and electrophysiological tests. In the particular case of spatial learning, when assessed using the Morris water maze, these deficits were found only in animals showing also hippocampal anatomical malformations (Szalkowski et al. 2012), suggesting a direct relationship between anatomical and behavioural changes. A similar result was obtained with animals electroporated with shRNA against *Dyx1c1* and subjected to the same experimental paradigm (Threlkeld et al. 2007). The lack of any obvious anatomical malformations in the *Kiaa0319* mutant mice could thus explain the lack of spatial learning and memory deficits in our model.

Interestingly, rats electroporated with shRNA against *Kiaa0319* and tested in the delayed match-to-sample radial water maze task showed no working memory deficit regardless of the presence or absence of hippocampal anomalies (Szalkowski et al. 2012). In contrast, knockdown of *Dyx1c1* in rats leads to defects in spatial working memory in the same test even in the absence of hippocampal malformations (Szalkowski et al. 2011). These results not only suggest a different role of *Kiaa0319* and *Dyx1c1* with regard to memory, but also that the lack of learning deficits that we report here might also be explained by the different tests used to assess learning and memory in our studies.

Even though some of the detailed analyses performed on shRNA electroporated rats cannot be carried out in mice, further testing with *Kiaa0319* ko mice might well uncover other behavioural changes that could shed light on the function of KIAA0319 in the developing and adult brain.

### Final considerations

Results obtained in rats using RNA interference against four key candidate dyslexia susceptibility genes (*DYX1C1*, *DCDC2*, *KIAA0319,* and *KIAA0319L*) are in agreement with the “neuronal migration” hypothesis of dyslexia but the studies conducted in mice have so far failed to provide solid evidence to support it. The *Dyx1c1* knockout mouse showed hydrocephaly and laterality defects, reminiscent of the phenotypes of mutants with defective motile cilia (Tarkar et al. 2013). Indeed, DYX1C1 is necessary for ciliary movement and its absence leads to primary ciliary dyskinesia in humans. Analysis of *Emx1-Cre/Dyx1c1* ^*flox/flox*^ conditional mutants found no defects in cortical lamination (Rendall et al. 2015) and the *Dcdc2* knockout did not show any migration defects (Wang et al. 2011). Interestingly, the product of the *Dcdc2* gene is localised to the primary cilium in hippocampal neurons, and its expression levels influence ciliary signalling (Massinen et al. 2011). *DCDC2* has recently been implicated in renal-hepatic ciliopathy, and histological changes consistent with the human phenotype have also been detected in the *Dcdc2* knockout mouse (Schueler et al. 2015). *DYX1C1*, *DCDC2,* and *KIAA0319* have been identified as novel potential cilia-related genes (Ivliev et al. 2012). This has led to speculations about a connection between the mechanisms that establish laterality and left/right asymmetry, and dyslexia (Brandler and Paracchini 2014). However, experimental evidence linking KIAA0319 to cilia is still missing.

While the available data from both shRNA treated rats and constitutive or conditional mouse knockouts clearly suggest a role for dyslexia susceptibility candidate genes in specific learning and auditory processing tasks, their hypothesised function in neuronal migration should be critically evaluated. The recent development of the CRISPR–Cas9 system has created the possibility of generating transgenic animals of species different than the mouse (Hsu et al. 2014). It would be interesting to assess whether the migration defects described in electroporated rat embryos can be reproduced by eliminating *Kiaa0319* expression through a different and more specific method. The results of such experiments would have special relevance when considering the role of *KIAA0319*, *DYX1C1,* and *DCDC2* in human corticogenesis.

Elucidating the molecular and cellular mechanisms underlying RD will continue to be a challenging task. Our results do not unveil major effects during brain development caused by removal of KIAA0319, adding evidence against the “neuronal migration” hypothesis of dyslexia. Interestingly, KIAA0319 has been identified as a novel player in axonal growth and regeneration, repressing the intrinsic growth capacity of axons (Franquinho et al. submitted). Therefore, the *Kiaa0319* knockout mouse characterised here should provide a new tool for a more in depth analysis into the putative role of this protein in brain development and function.

## Electronic supplementary material

Below is the link to the electronic supplementary material.

Supplementary material 1 (PDF 12406 kb)
