# Supplementary material for: Normal radial migration and lamination are maintained in dyslexia-susceptibility candidate gene homolog Kiaa0319 knockout mice
Source: Brain Struct Funct. 2016 Aug 10;222(3):1367–84. doi: 10.1007/s00429-016-1282-1 (PMC5368214; doi:10.1007/s00429-016-1282-1)

BRAIN STRUCTURE AND FUNCTION

SUPPLEMENTARY INFORMATION

Normal radial migration and lamination are maintained in dyslexia-susceptibility candidate gene homolog *Kiaa0319* knockout mice

Isabel Martinez-Garay[1,2], Luiz G. Guidi^[1,3], Zoe G. Holloway^[3], Melissa A. G. Bailey[1], Daniel Lyngholm[1,4], Tomasz Schneider[5], Timothy Donnison[3], Simon J. B. Butt[1], Anthony P. Monaco*[3,6], Zoltán Molnár*[1], Antonio Velayos-Baeza*[3]

1. Department of Physiology, Anatomy, and Genetics, University of Oxford, Oxford, UK
2. Current address: Division of Neuroscience, School of Biosciences, Cardiff University, Cardiff, UK
3. Wellcome Trust Centre for Human Genetics, University of Oxford, Oxford, UK
4. Current address: Strathclyde Institute of Pharmacy and Biomedical Sciences, University of Strathclyde, Glasgow, UK
5. School of Pharmacy and Biomedical Sciences, University of Central Lancashire, Preston, UK
6. Current address: Tufts University, Medford, USA

* Corresponding authors

A.V.-B.: Wellcome Trust Centre for Human Genetics, University of Oxford, Oxford, OX3 7BN, UK. Tel.: +44 (0)1865 287502. Fax.: +44 (0)1865 287501. Email: avelayos@well.ox.ac.uk

Z.M.: Department of Physiology, Anatomy, and Genetics, University of Oxford, Oxford, OX1 3QX, UK. Tel.: +44 (0)1865 282664. Fax.: +44 (0)1865 272420. Email: Zoltan.Molnar@dpag.ox.ac.uk

A.P.M.: Office of the President, Ballou Hall, Tufts University, Medford, MA 02155, USA. Tel.: 1 617 627 3300. Fax.: 1 617 627 3555. Email: anthony.monaco@tufts.edu

SUPPLEMENTARY MATERIALS AND METHODS

Generation of *Kiaa0319*-targeted mice

Mouse JM8.F6 embryonic stem cells (Pettitt et al. 2009) targeted at the *KIAA0319*-homologous mouse gene *D130043K22Rik* with a "knockout-first" (KO1, reporter-tagged insertion with conditional potential) allele (C57BL/6N-*D130043K22Rik*$^{tm1a(KOMP)Wtsi}$) (Skarnes et al. 2011), generated under project CSD26319, were obtained from the Knock-Out Mouse Project (KOMP) repository at UC Davis, California (www.komp.org). The acquired clones (EPD0073_1_C12, EPD0077_5_D04 and EPD0077_5_F04) had passed KOMP quality control tests and were confirmed to be around 75% euploid. These clones were used for albino C57BL/6J blastocyst injections at the Transgenics Core of the Wellcome Trust Centre for Human Genetics in Oxford (UK). Three male chimeras were obtained from *Kiaa0319-KO1* ES cell clone D04 (~ 90%, 80% and 30% contribution from the injected ES cells), and one with clone C12 (~20% contribution). Germline transmission was determined for the D04-chimeras by the presence of black coated pups in litters breeding of male chimeras with albino C57BL/6J females or by genotyping. This was successful for the 90% and 80% chimeras, and both males were initially used for backcrossing into C57BL/6J background, but only mice derived from the 80% chimera were kept and used in this work.
Subsequent C57BL/6J backcrossing was used to generate a "*Kiaa0319-KO1*" colony (C57BL/6J-*D130043K22Rik*$^{tm1a(KOMP)Wtsi}$). *Kiaa0319-NZ* mice (C57BL/6J-*D130043K22Rik*$^{tm1b(KOMP)Wtsi}$) carrying the *Kiaa0319* "Null-lacZ" (NZ) allele were obtained after removal of the promoter-driven *neo* cassette and floxed exon 6 by Cre-mediated recombination after crossing a *Kiaa0319-KO1* male with B6.Cg-Tg(Sox2-cre)1Amc females (Hayashi et al. 2002; Vincent and Robertson 2003). *Kiaa0319-Flx* mice (C57BL/6J-*D130043K22Rik*$^{tm1c(KOMP)Wtsi}$) carrying the *Kiaa0319* "floxed" (Flx) (conditional KO potential) allele were obtained after removal of the IRES:*lacZ* trapping cassette and promoter-driven *neo* cassette by FLPe-mediated recombination after crossing with B6.Cg-Tg(ACTFLPe)9205Dym/J mice (Rodriguez et al. 2000). *Kiaa0319-Null* mice (C57BL/6J-*D130043K22Rik*$^{tm1d(KOMP)Wtsi}$) carrying the *Kiaa0319* "Null" allele were obtained after removal

of the floxed exon 6 by Cre-mediated recombination after crossing a *Kiaa0319-Flx* male with Sox2-cre females. Information about gene organisation and details of the targeting strategy are provided in Fig. S1. A detailed diagram of the different alleles is shown in Fig. S2.
The expected effect of the trapping cassette in *KO1* and *NZ* alleles is to prevent normal splicing after exon 5, which will be joined to the *En2* exon present in the cassette, and resulting in a short chimeric protein (p.D374GfsX102) lacking the PKD, C6, transmembrane and cytoplasmic domains of KIAA0319 protein (Velayos-Baeza et al. 2007, 2008). The predicted result at the protein level from the *Null* allele is p.D374VfsX14 although the resulting transcript, unlike with the *KO1* and *NZ* alleles, would be expected to undergo degradation by nonsense-mediated mRNA decay (NMD) and no protein would be produced.
C57BL/6J mice were obtained from Harlan Laboratories UK. Both FLPe and Sox2cre mouse lines were already available at the WTCHG animal facility, kept by backcrossing into C57BL/6J.

Custom anti-KIAA0319 antibodies

Specific antiserum R7 against the C-terminal domain of mouse KIAA0319 protein has been previously described (Velayos-Baeza et al. 2010). Custom polyclonal rabbit antiserum R5 against the ectodomain of KIAA0319 was obtained from Eurogentec Ltd after immunisation with peptides [E+F] (residues 150-164, C+ PEETTEYSDEYKDLE, and 208-222, MEKLQDPTPHPLDQE+C, respectively, of the mouse KIAA0319 protein). Characterisation of reactivity in Western blotting and Immunofluorescence applications, including affinity-purified antibodies against each of the immunisation peptides and performed using overexpression of mouse and human proteins in mammalian cells, showed that only epitope F in the mouse protein was recognised and that only non-glycosylated protein seemed to be detected (results not shown). Therefore this antiserum is not able to recognise the endogenous, glycosylated, mouse KIAA0319 protein. However, transient overexpression of the KIAA0319 protein allows accumulation of non-modified protein that can be detected by the R5 antiserum.

Cortical neuron cultures, nucleofection, and processing. Cortical neuron cultures were performed as described in (Maximov et al. 2007) with minor modifications. Briefly, C57BL/6J E16.5 embryos were dissected and cortices digested in 6mg/ml trypsin (Sigma-Aldrich) at 37°C for 7 minutes. After inactivation of trypsin with serum-containing HBSS, tissue was dissociated using a P1000 tip. Following dissociation, $5x10^6$ cells/sample were nucleofected with plasmid DNA (4 µg) using the Amaxa Mouse Neuron Nucleofector system for primary Mouse Cortical Neurons (VPG-1001, program O-005). Transfected neurons were plated at $1.5x10^7$ cells/well on 90 mm plates pre-coated with 0.01% poly-L-lysine (Sigma-Aldrich) and 10 µg/ml laminin (Sigma-Aldrich), and cultured for 72 h at 37°C with 5%CO_2 in Neurobasal medium (Gibco) supplemented with 100 ug/ml insulin (Sigma-Aldrich), 100 ug/ml transferrin (Merck Chemicals), 1x B27 supplement (Gibco), 5% FBS serum (HyClone), 1% penicillin/streptomycin (Gibco) and 2 mM L-glutamine (Gibco). Cells were then collected after treatment with trypsin, washed with cold PBS, and used for DNA extraction (same protocol as described for ear notches) and protein lysate preparation by direct re-suspension in RIPA buffer (conditions as described in main text).

Behavioural tests

Elevated Plus Maze (EPM). The test was essentially carried out as previously described (Ufartes et al. 2013). The maze consisted of 2 opposing open and 2 opposing closed arms. All arms were 30 cm long and 5 cm wide. Half the mice were placed in the EPM facing left and the other half facing right in the open arm and allowed to freely explore the apparatus over a 10-min trial. Animals were tracked using the AnyMaze System (Stoelting, USA). Entry into an arm of the EPM was defined using the entire animal with at least 70% of the animal being in the zone. The percentage of time spent in the open arms was calculated by dividing the time spent in the open arms by the combined time spent in open and closed arms. The percentage of open arm entries was also calculated.

Open field. Locomotor activity was measured using the PAS Home Cage system (San Diego Instruments, San Diego, CA, US). Mice were placed into a plexiglass cage with 4x8

photobeam configuration and tested over a period of 60 min. The total beam breaks were measured and divided into 10 minute time bins for analysis.

Locomotor habituation. Locomotor habituation to the increasingly familiar environment was assessed using a computer controlled system (TSE-Systems, Bad Homburg, Germany). Mice were exposed 3 times for 10 minutes to the same rectangular Plexiglas cage [35 cm (w) × 20 cm (d) × 20 cm (h)] inserted into the TSE-System with 1 hour interval between exposures. Cages were cleaned with water and alcohol between animals. Animals' locomotor activity was automatically scored by the photobeam system (15x15 beams); decrease in distance travelled was used as a measure of habituation.

Rotarod. The acceleration rotarod was carried out as described (Ufartes et al. 2013). Mice were given 3 trials per session and 3 sessions per day with a 60-min intersession interval. Mean speed at fall from three consecutive trials was used as a measure of motor coordination. If a mouse failed to grip in three times in the initial 10 s/4rpm period it was excluded.

Inverted screen, Grip strength, Spatial novelty (Y maze) and Social behaviour / social memory. Tests were performed as described (Ufartes et al. 2013).

Spontaneous alternations (T maze). The apparatus and method have been described (Ufartes et al. 2013). Each mouse received 6 trials over 2 sessions. The inter-session interval was 80 minutes. The percentage of passes over 6 trials was calculated to indicate the degree to which the mouse explored a different goal arm to the previous run (alternation).

Object recognition. Med Associates activity chambers (ENV-510) were used containing a black Perspex box (27 x 27 cm) insert. The objects were metal brackets glued to 5 x 5 cm metal bases and novel and familiar objects were similar in size, approximately 6.5-8.5 cm x 3.5-4 cm. During testing, the objects were placed 4 cm from the middle part of the walls to allow investigation around the object. Tests were recorded and analyzed by the AnyMaze System (Stoelting, USA). The test consisted of three sessions. During session 1 (habituation phase) the mouse was allowed to freely explore in an empty open field arena for 10 minutes

and locomotor activity was assessed. During session 2 (exposure phase), two identical objects were placed in the arena and the mouse freely allowed to investigate the objects. Response to object novelty was examined during session 3 (test phase) by replacing one of the familiar with a novel object and the mouse was allowed to explore. Session 2 and 3 were 5 min in duration and were separated by a 3-min inter-trial interval (ITI) where the mouse was placed in a holding cage next to the testing box. During this period, the box and objects were wiped with water and 70% alcohol. The duration and frequency of object exploration during each session was recorded and response to object novelty was assessed by comparing the time spent in contact with the novel object versus the time spent in contact with the familiar object. Object exploration was scored only when the mouse's head was in contact with the object. The position of the novel and familiar objects were counterbalanced. A performance ratio was calculated (novel/novel+familiar) where 0.5 denotes equal object preference whilst higher or lower denotes a preference for novel or familiar respectively.

Light/dark box. The test was performed as previously described (Scheneider et al. 2012) in Med Associates activity chambers (ENV-510). The dark box insert was made of black Perspex designed to cover half the area of the activity chamber (27 x 13.9 x 21.5 cm) with a 4 x 4 cm hole placed in the middle of the wall at floor level. Time spent in and latency to enter light and dark zones as well as the number of full-body transitions between the light (300 lux) and dark (2 lux) compartments were automatically scored by Med Associates activity software. Animals were started in the light compartment; the session lasted 10 minutes.

Pre-pulse inhibition of acoustic startle. Startle response and pre-pulse inhibition of acoustic startle responses were measured by the SR-Lab System (San Diego Instruments, San Diego, CA, USA). The mouse was placed in a Plexiglas cylinder and left to acclimatise for 5 min with constant 65dB sound (background noise). A test session contained ten periods in which the trial stimuli were included in pseudorandom order so that they appeared once within each period of 12 trial stimuli: startle stimulus (40 ms, 120 dB sound burst), pre-pulse stimulus (20 ms, 81 dB), baseline stimulus (20 ms, 65 dB, to measure baseline movement in

the cylinders), and 9 combinations of pre-pulse and startle stimuli (“pre-pulse-plus-pulse”) spaced by either 10, 20, 30, 40, 50, 100, 200, 400, 800 ms delays, starting at the end of the pre-pulse stimulus. The test session started and finished with five startle stimuli. The average inter-trial interval was 15 s (ranged from 10 to 20 s). The startle response was recorded every 1 ms during a 65 ms sampling window starting with the onset of the startle stimulus or the 81dB pre-pulse alone stimulus. The following formula was used to calculate the percentage of pre-pulse inhibition of a startle response: 100–(“pre-pulse-plus-pulse” ×100/”pulse-alone”).

Unistat 6.5 (Unistat Ltd, London, UK) software was used to perform statistical data analysis using one way one or two-way analysis of variance (ANOVA). To follow up, post-hoc analysis using Bonferroni-modified least significant difference was used. When animals were tested multiple times in the same task or within session periods were used, ANOVA with repeated measures was applied.

SUPPLEMENTAL REFERENCES

Hayashi S, Lewis P, Pevny L, McMahon AP (2002) Efficient gene modulation in mouse epiblast using a Sox2Cre transgenic mouse strain. Mech Dev 119 Suppl 1:S97-S101. doi: 10.1016/S0925-4773(03)00099-6

Lambert JF, Benoit BO, Colvin GA, et al (2000) Quick sex determination of mouse fetuses. J Neurosci Methods 95:127-132. doi: 10.1016/S0165-0270(99)00157-0

Maximov A, Pang ZP, Tervo DGR, Sudhof TC (2007) Monitoring synaptic transmission in primary neuronal cultures using local extracellular stimulation. J Neurosci Methods 161:75-87. doi: 10.1016/j.jneumeth.2006.10.009

Pettitt SJ, Liang Q, Rairdan XY, et al (2009) Agouti C57BL/6N embryonic stem cells for mouse genetic resources. Nat Methods 6:493-495. doi: 10.1038/nmeth.1342

Rodriguez CI, Buchholz F, Galloway J, et al (2000) High-efficiency deleter mice show that FLPe is an alternative to Cre-loxP. Nat Genet 25:139-140. doi: 10.1038/75973

Vincent SD, Robertson EJ (2003) Highly efficient transgene-independent recombination directed by a maternally derived SOX2CRE transgene. Genesis 37:54-56. doi: 10.1002/gene.10226

SUPPLEMENTARY FIGURE LEGENDS, TABLES AND FIGURES

Fig. S1 *Kiaa0319* gene organisation and targeting strategy. **a** Details of gene organisation, obtained from Ensembl (www.ensembl.org). Exon 6 is targeted with a “knock-out first” cassette (KOMP project CSD26319). **b** The regions present in the different alleles are described, showing start and end positions using first position of the start codon as position +1

Fig. S2 Generation of *Kiaa0319*-targetted mice. **a** Detailed schematic representation of strategy followed for targeting the *Kiaa0319* gene. Two regions flanking exon 6 were replaced by a trapping cassette (containing two FRT sites (F1, F2) and two loxP sites (P1, P2)) and a loxP-cassette (containing a third loxP site (P3)), respectively, to obtain a “knock-out first” (*tm1a* or KO1) allele. After recombination with Cre, the region between sites P1 and P3 is deleted to obtain a “Null-lacZ” (*tm1b* or NZ) allele. Deletion of region between sites F1 and F2 by Flp recombination generates a “floxed” (*tm1c* or Flx) allele with conditional knock-out potential: a “Null” (*tm1d* or Null) allele where exon 6 is deleted can be generated after Cre recombination between sites P2 and P3. Long-range PCR fragments (KO1-1 to KO1-4) used for target confirmation and sequencing, and the PCRs used for identification of the different alleles (genotyping) are shown. Details about elements in the targeting cassette and the different PCRs are shown in Fig. S3 and S4. **b** Results of long-range PCRs from KO1 homozygous mice obtained from two different male chimeras (80% and 90%). DNA ladder sizes are shown on the right. **c** Results obtained with genotyping PCRs from mice homozygous for wild type (ww), KO1 (aa), NZ (bb), Flx (cc) or Null (dd) alleles; heterozygous Flx (cw) also included to show double band with KWF. Size of each fragment (bp) is shown on the right. **d** Western blotting analysis from different organ lysates from 6-week old mice with specific antiserum R7 shows a heterogeneous band pattern in wild type (wt) samples (left panel). The same pattern is maintained in samples from homozygous NZ mice (middle and right panels) except for a band of ~170kDa (*white asterisk*), the expected size for

glycosylated full-length KIAA0319 protein, in brain and cerebellum (and possibly testis) samples. 30 µg (left panel) or 50 µg (middle and right panels) total protein loaded per lane. Br, brain; Li, liver; Ki, kidneys; Sp, spleen; Lu, lungs; He, heart; Te, testis

Fig. S3 Sequence of *Kiaa0319-KO1* allele. The region between primers GF1 and GR1 of the "knockout first" allele for *Kiaa0319* gene is provided. Important regions, elements of the targeting cassette and primers used for genotyping and PCR amplification (see Fig. S2 and Table S1) are shown. Forward primers are underlined and named on the left side; reverse primers are in bold and named on the right side; name of primers used for amplification of the overlapping PCR fragments KO1-1 to KO1-4 are colour-highlighted. After sequencing these long-range PCR fragments we only found 3 differences (*yellow highlighted*) with the expected sequence, none of which affected any critical element: an extra A at the IRES element, an extra G at the b-actin promoter (position -354/5 upstream the Neo resistance gene), and an extra G in intron 6 (position +151/2) of the *Kiaa0319* gene

Fig. S4 PCR and genotyping details. Primer combinations and expected fragment sizes for long-range, genotyping and sex-determination PCRs used in this work, as well as the general genotyping amplification profile, are described. Schematic representation of genotyping results for each PCR is presented for each possible genotype for the *Kiaa0319* locus; (+), amplification; (-), no amplification; when two fragments can be amplified, the larger one is denoted as [+]

Fig. S5 Normal lamination in early developmental stages. **a** Immunostaining of cortical sections of *Kiaa0319* +/+, +/- and -/- embryos at E15.5 with anti-Ctip2 (green). **b** Immunostaining of cortical sections of *Kiaa0319* +/+, +/- and -/- embryos at E18.5 with anti-Ctip2 (green) and anti-Cux1 (red). No differences are detected between the three genotypes. Nuclei were stained with DAPI (blue). VZ, ventricular zone; SVZ, subventricular zone; IZ, intermediate zone; CP, cortical plate. Scale bars = 75 µm

Fig. S6 Generation of *Null* allele by Cre expression in *Flx* electroporated cells. **a** Representative immunostaining image showing expression of Cre in transfected cells. Embryos from homozygous (F/F) floxed animals were electroporated *in utero* at E14.5 with pCIG-Cre. Transfected cells express EGFP (green) and stain with an anti-Cre antibody (red). Scale bar = main image, 100 µm; insets, 50 µm. **b** Detection of *Kiaa0319 Flx* (*tmc1*, F) and/or *Null* (*tmd1*, N) alleles with PCR KNF (see Fig. S2c). Primary neuronal cultures from homozygous (F/F) floxed embryos were transfected *in vitro* with pCIG-Cre (CRE) or the control plasmid pCIG (cntr). *Null* allele is detected in the Cre-transfected culture but not in control, where only *Flx* allele is detected. DNA samples from heterozygous *Null* (wt/N) and homozygous *Flx* (F/F) mice were used as controls. **c** Western blotting analysis of neuronal cultures described in **b** shows a reduced level of KIAA0319 protein (*arrow*) in Cre-transfected cells. Analysis of brain lysates from wild type (wt/wt), heterozygous (wt/N) and homozygous (N/N) *Null* mice at postnatal day 2 (P2) confirms the expected knock-out effect of this allele, with KIAA0319 protein completely absent in homozygous N/N mice. Lower bands are unspecific signal as shown in Fig. 1 and Fig. S2d

Fig. S7 Behavioural characterisation of *Kiaa0319* mutant mice. No difference in performance was observed between *Kiaa0319* homozygous (-/-) or heterozygous (+/-) and wild type (+/+) controls in a number of locomotor, strength and learning and memory tests: **a** locomotor activity in open field; **b** social approach task, preference for social novelty; **c** locomotor habituation; **d** inverted screen; **e** weight lifting; **f** motor coordination and learning in rotarod; **g** spontaneous alternations in T maze; **h** spatial novelty in Y maze; **i** object recognition. All data shown are mean ± s.e.m. Number of animals analysed for each genotype per test as shown in Table S3

Table S1 Primers used in this work

Primer Name	Primer Sequence	Reverse complementary to	location	Start position in *Kiaa0319* alleles / comments				
				Wild type	KO1 (tm1a)	Null-Z (tm1b)	Floxed (tm1c)	Null (tm1d)
mKi2F3 (GF3)	CATGTCATGTGTGTGCATGCATATATGTGC		intron 2	1825	1825	1825	1825	1825
mKi2F4 (GF4)	GTCATGTGTGTGCATGCATATATG		intron 2	1828	1828	1828	1828	1828
mK3F2 (GF2)	CTACAGCTACAGGAGACAACTCAG		exon 3	2686	2686	2686	2686	2686
mKi3F1 (GF1)	CAGACTCTTTCAGGCTAGAGGAAC		intron 3	2988	2988	2988	2988	2988
mK5F	TTGCTGTGTCTGCTGGAGAT		exon 5	8147	8147	8147	8147	8147
mKi5F1	GCATTCTCTGAGCCACTACC		intron 5	8284	8284	8284	8284	8284
SA-F1	AAGGCGCATAACGATACCAC		5'FRT(1)		8618	8618	8618	8618
LAR3	CACAACGGGTTCTTCTGTTAGTCC	GGACTAACAGAAGAACCCGTTGTG	En-2 intron		8821	8821		
lacZ-F3	AATCGCCTTGCAGCACATCC		lacZ		10443	10443		
lacZ-R3	GTTCAACCACCGCACGATAG	CTATCGTGCGGTGGTTGAAC	lacZ		11313	11313		
lacZ-F8	AATCAGGCCACGGCGCTAAT		lacZ		11752	11752		
lacZ-R5	GACATCCAGAGGCACTTCAC	GTGAAGTGCCTCTGGATGTC	lacZ		12386	12386		
lacZ-F1	TGGCGAGCGATACACCGC		lacZ		12985	12985		
lacZ-R7	AGGAGTCGTCGCCACCAATC	GATTGGTGGCGACGACTCCT	lacZ		13365	13365		
lacZ-F2	GCCCGTCAGTATCGGCGG		lacZ		13369	13369		
PNF	ATCCGGGGGTACCGCGTCGAG		5'FRT(2)		15566			
R2R	TCTATAGTCGCAGTAGGCGG		3'loxP(2)		15720		8816	
mKi5F3	CGGACCTAGTGAGGTTGAAG		target, intron 5	8679	15743		8839	
mKi5R3	GAATCTCCGGTCTCTGTGTC	GACACAGAGACCGGAGATTC	target, intron 5	8961	16025		9121	
mKi6R1	GCAGGCACATGCCATCCATC	GATGGATGGCATGTGCCTGC	target, intron 6	9318	16382		9478	
loxP3R	GATGGCGAGCTCAGACCATA	TATGGTCTGAGCTCGCCATC	3'loxP(3)		16507	13779	9603	8786
mKi6R2	CATCCATCTGCCTGATTGAG	CTCAATCAGGCAGATGGATG	intron 6, replaced	9463				
mKi6R3	GGAGCCAAGGAAGTGGTATG	CATACCACTTCCTTGGCTCC	intron 6	9711	16744	14016	9840	9023
mKi6R4	CAATGACGTGAGCTGGTAAG	CTTACCAGCTCACGTCATTG	intron 6	9803	16836	14108	9932	9115
mKi8R9 (GR2)	CCAGTAGTGGAGAAAGAGGCTGTG	CACAGCCTCTTTCTCCACTACTGG	intron 8	14657	21690	18962	14786	13969
mKi8R10 (GR4)	GTGGTACAGTGCTTAACTTCCATGCATGTG	CACATGCATGGAAGTTAAGCACTGTACCAC	intron 8	14705	21738	19010	14834	14017
mKi8R11 (GR1)	GCACGTTGAAGTCACGTTGAAGAG	CTCTTCAACGTGACTTCAACGTGC	intron 8	15907	22940	20212	16036	15219
mKi8R12 (GR3)	GACTAGACACTCACCTTGGTGACATGC	GCATGTCACCAAGGTGAGTGTCTAGTC	intron 8	16084	23117	20389	16213	15396
Cre-F2	GGTCGATGCAACGAGTGATG		Cre					
Cre-F3	AGGCGTTTTCTGAGCATACC		Cre					
Cre-R2	GATCCGCCGCATAACCAGTG	CACTGGTTATGCGGCGGATC	Cre					
Cre-R5	TCGGATCATCAGCTACACCA	TGGTGTAGCTGATGATCCGA	Cre					
Cre-R6	TCCAGACCAGGCCAGGTATC	GATACCTGGCCTGGTCTGGA	Cre					
AGp-F2	AGAGCCTCTGCTAACCATGT		Intron, AG promoter	ß-actin/ß-globin promoter, between Sox2 enhancer/promoter and Cre				
oIMR7338	CTAGGCCACAGAATTGAAAGATCT		IL2, exon 3	Internal Positive Control, JAX Tg(ACTFLPe)9205Dym protocol				
oIMR7339	GTAGGTGGAAATTCTAGCATCATCC	GGATGATGCTAGAATTTCCACCTAC	IL2, intron 3	Internal Positive Control, JAX Tg(ACTFLPe)9205Dym protocol				
oIMR1348	CACTGATATTGTAAGTAGTTTGC		FLP1	FLP1, +348 to +370, JAX Tg(ACTFLPe)9205Dym protocol				
oIMR1349	CTAGTGCGAAGTAGTGATCAGG	CCTGATCACTACTTCGCACTAG	FLP1	FLP1, +1072 to +1051, JAX Tg(ACTFLPe)9205Dym protocol				
mSry-F1	ATACAGAGATCAGCAAGCAGC		Sry, exon 1	Mouse Sry locus, +95 to +115				
mSry-R1	GTGTGCAGCTCTACTCCAGTC	GACTGGAGTAGAGCTGCACAC	Sry, exon 1	Mouse Sry locus, +366 to +346				
mSry-F	TGGGACTGGTGACAATTGTC		Sry, promoter	Mouse Sry locus, -28 to -9 (Lambert et al. 2000)				
mSry-R	GAGTACAGGTGTGCAGCTCT	AGAGCTGCACACCTGTACTC	Sry, exon 1	Mouse Sry locus, +374 to +355 (Lambert et al. 2000)				
mIL3-F	GGGACTCCAAGCTTCAATCA		IL3, exon 1	Mouse IL3 gene, 792–801 (Lambert et al. 2000)				
mIL3-R	TGGAGGAGGAAGAAAAGCAA	TTGCTTTTCTTCCTCCTCCA	IL3, intron 2	Mouse IL3 gene, 1335–1316 (Lambert et al. 2000)				
mKiaa-F-XhoI	AAGCTCGAGGTGAGGCAGAATGGTGTCCCCAC			for generation of pCIG-mKiaa0319, from position -10				
mKiaa-R-EcoRI	CATGAATTCTTACTATCTGTCCTTTGAGTAGTAACCG	CGGTTACTACTCAAAGGACAGATAGTAAGAATTCATG		for generation of pCIG-mKiaa0319, until stop codon				
Cre-F-XhoI	ACTCTCGAGGAGAACCACCATGGGCCCAAAG			for generation of pCIG-Cre				
Cre-R-EcoRI	CCAGAATTCTCACTAATCGCCATCTTCCAGCAG	CTGCTGGAAGATGGCGATTAGTGAGAATTCTGG		for generation of pCIG-Cre				

All primers used in PCR, sequencing and cloning are described; for "reverse" primers, the reverse complementary sequence is also provided

Primers in genomic region around mouse *Kiaa0319 (D130043K22Rik)* exon 6 in different alleles are shown in the top section of the table; these primers are also described in Figure S2

For each primer, the position of their fisrt nt in the different *Kiaa0319* alleles (taking A in ATG condon as position +1) is shown

Primers described in KOMP project CSD26319 appear in red

Table S2 Primary antibodies used in Immunostaining experiments

Antibody	Species	Dilution	Company	Cat.#
anti-Pax6	rabbit polyclonal	1:200	Covance	PRB-278P
anti-TBR2	rabbit polyclonal	1:500	Abcam	ab23345
anti-pH3 (Histone H3 (phospho S10))	mouse monoclonal	1:2000	Abcam	ab14955
anti-Ki67	rabbit polyclonal	1:1000	Abcam	ab15580
anti-Ctip2 [25B6]	rat monoclonal	1:500	Abcam	ab18465
anti-Cux1 [CDP (M-222)]	rabbit polyclonal	1:200	Santa Cruz Biotechnology	sc-13024
anti-NF-H (Neurofilament H) [SMI32]	mouse monoclonal	1:2500	Covance	SMI-32P
anti-Calbindin D-28k	rabbit polyclonal	1:2000	Swant	CB38
anti-NeuN [A60]	mouse monoclonal	1:100	Chemicon	MAB377
anti-KIAA0319 [R5]	rabbit polyclonal	1:1000	Custom	This work

Table S3 Number of mice used per test in behavioural analysis

Test	Males			Females			
	**WT**	**Het**	**Hom**	**WT**	**Het**	**Hom**	**Total**
**Elevated Plus Maze**	8	9	8	9	8	6	**48**
**Open Field**	8	9	8	9	8	6	**48**
**Locomotor Habituation**	8	9	8	9	8	6	**48**
**Rotarod**	8	8	6	9	8	6	**45**
**Inverted Screen**	8	9	8	9	8	6	**48**
**Grip strength / Weight Lifting**	8	9	8	9	8	6	**48**
**Spontaneous Alternations (T-maze)**	8	9	8	8	8	6	**47**
**Spatial Novelty (Y-maze)**	8	9	8	8	8	6	**47**
**Object Recognition**	8	9	8	8	8	6	**47**
**Light/Dark Box**	8	9	8	8	8	6	**47**
**Social Behaviour**	8	9	8	8	8	6	**47**
**Auditory Sensorimotor Gating (Prepulse Inhibition)**	7	8	7	7	4	6	**39**

Note: Maximum number of animals appears in top row. Lower numbers, shown in blue, were used when animals died (1 wt female) or were excluded from analysis (3 mice excluded for Rotarod after failing the initial grip test; 8 animals excluded for Auditory Sensorimotor Gating for being deaf (score less than 50 in reaction to 120 dB))

Fig. S1 Structure of mouse Kiaa0319 (D130043K22Rik) gene and generation of targetted alleles

a

**Genome assembly:**	**GRCm38.p4 (GCA_000001635.6)**
**MGI transcript name:**	**D130043K22Rik-001**
**Gene**	**RIKEN cDNA D130043K22 gene [MGI:3036268]**
**Ensembl Gene**	ENSMUSG00000006711
**Location**	Chromosome 13: 24,845,135-24,901,270
**Ensembl Transcript**	ENSMUST00000006893
**Ensembl Protein**	ENSMUSP00000006893
**Strand**	Forward
**Base pairs**	4,973 — **24,854,501** ATG codon start position (exon 2)
**Amino acids**	1,081

No.	Exon / Intron	Start	End	Phase Start	Phase End	Length	Length	region	Size in allele WT	Size in allele KO1
	5' upstream sequence									
1	ENSMUSE00000470454	24,845,135	24,845,299	-	-	165				
	Intron 1-2	24,845,300	24,854,409			9,110				
2	ENSMUSE00000251843	24,854,410	24,854,555	-	1	146				
	Intron 2-3	24,854,556	24,856,651			2,096				
3	ENSMUSE00000251832	24,856,652	24,857,427	1	0	776				
	Intron 3-4	24,857,428	24,857,920			493				
4	ENSMUSE00000251824	24,857,921	24,858,110	0	1	190				
	Intron 4-5	24,858,111	24,858,122			4,527	12			
		24,858,123	24,862,637				4,515	5'arm	4,995	4,995
5	ENSMUSE00000251814	24,862,638	24,862,736	1	1	99	99			
	Intron 5-6	24,862,737	24,863,117			857	381			
		24,863,118	24,863,175				58		58	7,122
		24,863,176	24,863,593				418	target	694	694
6	ENSMUSE00000251805	24,863,594	24,863,691	1	0	98	98			
	Intron 6-7	24,863,692	24,863,869			1,041	178			
		24,863,870	24,863,980				111		111	80
		24,863,981	24,864,732				752	3'arm	5,094	5,094
7	ENSMUSE00000251799	24,864,733	24,864,820	0	1	88	88			
	Intron 7-8	24,864,821	24,867,088			2,268	2,268			
8	ENSMUSE00000117107	24,867,089	24,867,181	1	1	93	93			
	Intron 8-9	24,867,182	24,869,074			4,072	1,893			
		24,869,075	24,871,253				2,179			
9	ENSMUSE00000251779	24,871,254	24,871,386	1	2	133				
	Intron 9-10	24,871,387	24,872,218			832				
10	ENSMUSE00000571452	24,872,219	24,872,447	2	0	229				
	Intron 10-11	24,872,448	24,875,908			3,461				
11	ENSMUSE00000117117	24,875,909	24,876,032	0	1	124				
	Intron 11-12	24,876,033	24,877,934			1,902				
12	ENSMUSE00000117119	24,877,935	24,878,067	1	2	133				
	Intron 12-13	24,878,068	24,879,595			1,528				
13	ENSMUSE00000117112	24,879,596	24,879,744	2	1	149				
	Intron 13-14	24,879,745	24,880,769			1,025				
14	ENSMUSE00001227905	24,880,770	24,880,921	1	0	152				
	Intron 14-15	24,880,922	24,882,540			1,619				
15	ENSMUSE00000117111	24,882,541	24,882,679	0	1	139				
	Intron 15-16	24,882,680	24,883,740			1,061				
16	ENSMUSE00001210728	24,883,741	24,883,900	1	2	160				
	Intron 16-17	24,883,901	24,885,577			1,677				
17	ENSMUSE00001304380	24,885,578	24,885,720	2	1	143				
	Intron 17-18	24,885,721	24,887,835			2,115				
18	ENSMUSE00001291388	24,887,836	24,887,958	1	1	123				
	Intron 18-19	24,887,959	24,889,823			1,865				
19	ENSMUSE00001299407	24,889,824	24,889,914	1	2	91				
	Intron 19-20	24,889,915	24,893,360			3,446				
20	ENSMUSE00001205600	24,893,361	24,893,452	2	1	92				
	Intron 20-21	24,893,453	24,899,620			6,168				
21	ENSMUSE00000780261	24,899,621	24,901,270	1	-	1,650				
	3' downstream sequence									

24,899,799 Stop codon end position (exon 21)

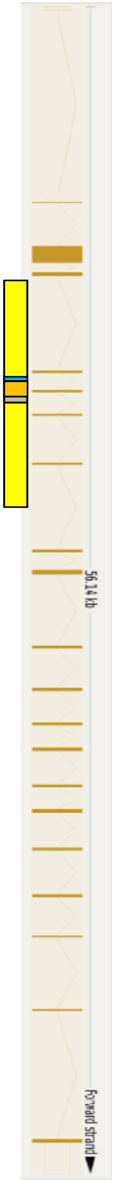

b

(Start of ATG codon is position +1)

Regions in different alleles		Size	WT Start	WT End	KO1 (tm1a) Start	KO1 (tm1a) End	Null-Z (tm1b) Start	Null-Z (tm1b) End	Floxed (tm1c) Start	Floxed (tm1c) End	Null (tm1d) Start	Null (tm1d) End		
5' to 5' homology arm				3622		3622		3622		3622		3622		
5' homology arm		**4995**	3623	8617	3623	8617	3623	8617	3623	8617	3623	8617		
"I5" (replaced intron 5 fragment)		**58**	8618	8675										
**SA-ßgeo-pA cassette (7122bp total)**	5'end	**64**			8618	8681	8618	8681	8618	8681	8618	8681		
	**FRT(1)**	**48**			8682	8729	8682	8729	8682	8729	8682	8729		
	En2-SA-IRES-lacZ-pA	**4999**			8730	13728	8730	13728					**a to c 6904bp removed**	
	**loxP(1)**	**34**			13729	13762								**a to b 2728bp removed**
	bAp-Neo-pA	**1823**			13763	15585								
	**FRT(2)**	**48**			15586	15633								
	SalI	**6**			15634	15639			8730	8735	8730	8735		
	**loxP(2)**	**34**			15640	15673			8736	8769			**c to d 817bp removed**	
	3'end	**66**			15674	15739			8770	8835				
**critical region (including exon 6)**		**694**	8676	9369	15740	16433			8836	9529				
**loxP cassette (80bp total)**	5'end	**23**			16434	16456			9530	9552				
	**loxP(3)**	**34**			16457	16490	13729	13762	9553	9586	8736	8769		
	3'end	**23**			16491	16513	13763	13785	9587	9609	8770	8792		
"I6" (replaced intron 6 fragment)		**111**	9370	9480										
3' homology arm		**5094**	9481	14574	16514	21607	13786	18879	9610	14703	8793	13886		
3' to 3' homology arm			14575		21608		18880		14704		13887			

Fig. S2 Generation of *Kiaa0319*-targeted mice

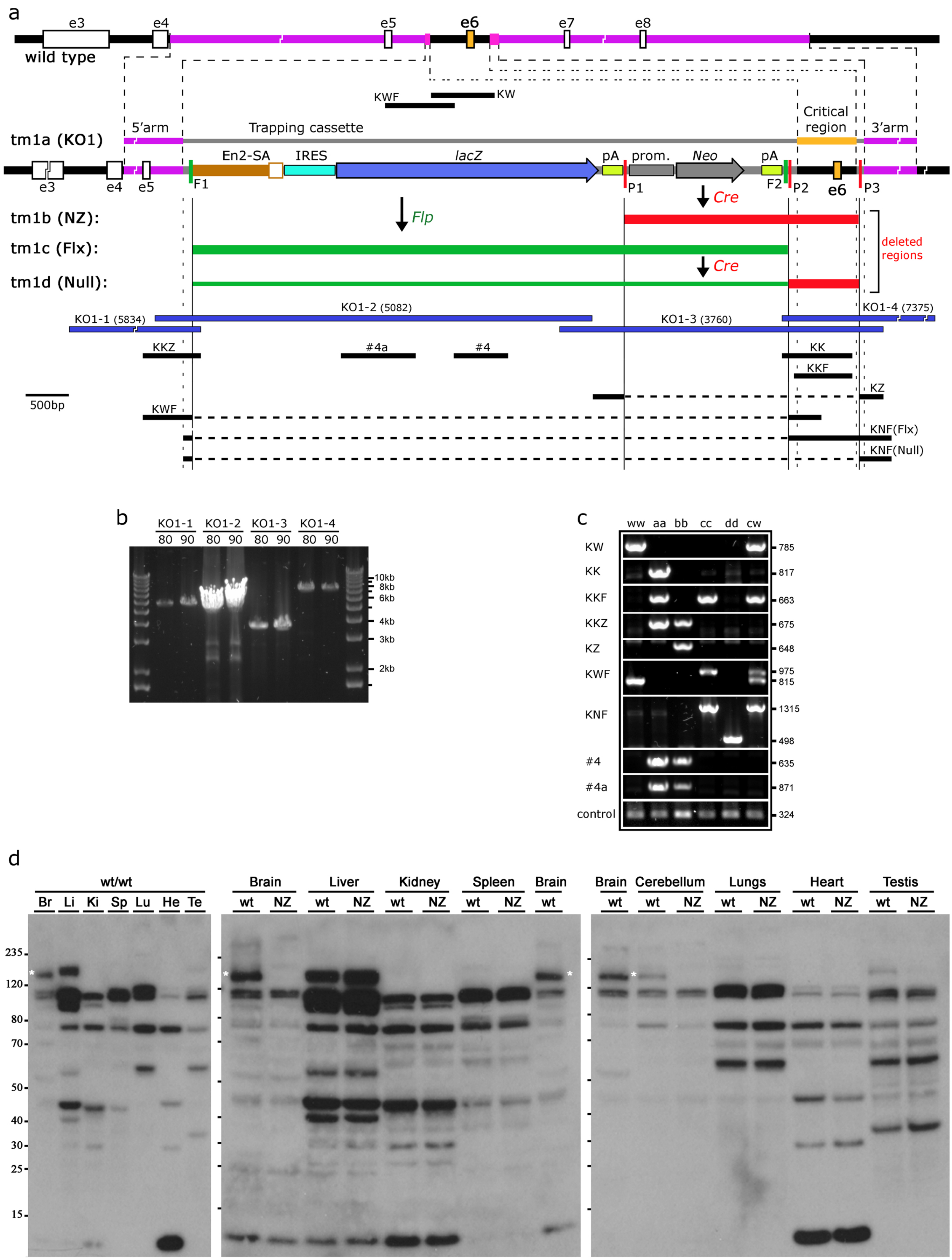

Fig. S3 *Kiaa0319 KO1* allele – Sequence between GF1 and GR1 primers

```
GF1 CAGACTCTTTCAGGCTAGAGGAACCCAAGGAGGTGTCTCCTAAAGGGCACTACCTCAGGCCGATGCCAGAGGAGGAATGT
CTGTCAGAGCCTCACCGGAAACAGAACAGCATTCTTTTGTTGTGCTTCCTTCTCTAAGCAGGCAGTTTGGAAAGAACACA
GAGCTTCCTCTTCCTAGGTTATCTACCTAAGAGTACCCGTTGAGGACACTAATTATAGGATCCTGTACCACCTCCCAGCT
CTGTCTTCTTGTAAGGAAGGCATTGGTACTGTTCTTGAGTGTTGTCACCCTGATGACCTGACCTCTACCTGTCTCTACCT
CCCCCTTTTAAGTGCACGGATTTGGAGACGGTGGGCACAAACAATCCATTGCTAAGCCTTCCTAGTCTTCATTTTTTTCC
ATAACAATATACCCTTGTTAATTCATCCCACAGGTTCCAATGCCTTCCCATAATCCTTCCCCTGCCAGCCTGGAGTCTAG
CCCAGCCACGACGGAAAAAAACTCCAACTTTACAGTCACGCCACGGAGCAGAAAACACAGCACCCCAACTTTTCCTACCA Exon 4
GTACAGTCCTCACTGGGCTAACCCCTCCTCCGTGGCCCTTGTCACCTACTGCTTCCAGGACAGGTAACTTCTGAAAACTG
TAATTAGAAGACACGTAAAGAATCTAGCAGCGACAATGTTTAAGGTTTTAGTCCTCCCTTCGGCCCCAGCCTTGAGGCAT
AACAGTCACAGTGCTATATAACCAAAGGGAAAAGATGAATTGCACTCGGCCTTGTCCCTCTGTGGCTCTGCAGAGCATGA
GAGGAGACTGACAGGTGGAAAGATGAGGCTGATACTCAGTGGGAAGTGGCGTGAGAGGATGCTCCGGCAGCAGAGGAAGA
TCGCCTGACCTCCCAGGAGTGCCAGGAAGCCTTTCTGGAGGAGGGACTGGCAGGTTAGGGTTAACCAGCTCACATGGGTC
TAGCCTGTTCTATAGTGGGAGCAGCGCATGCTCTAACGGGACGGAGTGTTGTATATATTTCTGGTTAATGAACTTCTAGC
AAGAACACAGGGCAGCTGTATTTCTATTTGAGGTGATCTCAATGTGGAGATAAGGGAACTGTAGAAAGCCTGTCCTGATG
CTGGAAGCAGAAGAAACATGACAGGTCAGAAGGGCCTGCAGTGTTCTCTAGTTTCTAATGCTTCTCTCCATGGGAAAGCA
CTGAGAGACTGTTGTTTCTGCATCTCAGCACCATGAGTGATCTCTTGCATCATGTGTGGAAGAGAGGTCAAGATTCACAA
CATTTTGGAAGAACCAGGAGATTGGTTGGCTAGAGCTGGTTTTGTGAGTAGAGAGGGCAAGATAGGAGTCTAGAATAGGA
AGGGTTGCCTAGATTAGTGCCAGGAGCCGTTAAGGAAAAGCTAAAAACTAGCAAGTGATCTTCTTGAGATGGTCCCACCA
TGGACTATACAGTTAATGATGGATTTGCTTCTTCAAACAAGGCAGAGGAGATGTGATTTTAAGAAAGTTTCCTGCTATTA
ATATTCTTTGCCTGTTATTATTAAAAATGTATAACATTCACTAAGTATTAGCAGATTTTGAGCAGATTTCTGCAGAACAA
CAAAAATGTACTGTCTTGAGTGATGCTATATAGACAAATAGATGATTTCTTAATTCTTAATTATGATTCTATAAGAATTC
CTAAAATTATGTTAGTAATTATTGAGCTCTTTTATAATAGGACTGCTTTTAAGTCCTTTCTGATGTCAAAAGTACATTGA
GAACTGTGCCAGTCTTCAAATGTCACCAGTTAATTGCTTCTGAGATAGCATGAAGGCATCTGTGACTTAGAGCACATGCC
AAGAAGTTGTAAAACAATAAACTAAAGGTCATACAAAGGGAATTGTGGACTTACTCTAGGTGCAAGGACAGAAGATAAAA
TATTGACTAGGTTTATCTATACAAAACTTCACTAATAACTTAGTCACAAAAGTTATGGTTTTTAATTCTCCATGAACTTG
TAGTGCTGGTGGCAGATGATGAATGTTTAGCCAGATAATTACTCATAATGGATATGCATATAAACCTTCTCATTTTAAAC
TTCCTACTCAATTTATGATTTGAATTTATGTTTAAACTTCTAGTGAACTTTTGTAAAAAAGTGGTGCTTGGAAAAAATCAT
GTGATCTGTGCTCCTAGAAAAGGTACAAAAAGACTAAGAAAGACTACATTGGACGTTATAATATTGAAGAGATAGCATTT
GGGAACATAGCATTGGGAGCAAGGCATTGGGAGTGAGGGCATTGTTACATCAGAGCAGGAGGAGAGAGCACAATTAGGAG
GAGAATGCAGAGTGGAATAACTTAAAAATATAGCCTCATGGGGTGTTCCCTATGATCGACTGACCGAAGAGGAGAAGACT
AGAGCCTGGTTTACTGATGGCTCTGCACGTTATGCAGGCACCACCCAGAAGTGGACAGCTGCAGCATTACAACCCCTTTC
TGGGACAACCTTGAAAGACACAGGTGAAGGGAAATCTTCACAGTGGGCAGAACTTCGGGCAGTACACATGGTATTACAGT
TTGTTTGCAAGAAGAAGTGGCCAGATGTACAATTATTCACTGACTCATGGGCTGTAGCCAATGGATTGGCTGGATGGTCA
GGCACTTGGAAAGATCACAATTGGAAAATTGGAAGAAAGGCAACTTAAAATATTAAGAGCAATATATAATGCAGAGGGAA
AGAAGAAAAAAGAAGAAGCTGTAGAGAGAGCAGAAGGAGCAGGAAGGCTTCTCCTTACCATGGGACAGAACAGTTCTTTT
CTTAAAAACAAGGCAGGTTTAGTCTTACTAAAGGGAACAAAGCTTTCTTCTTACAGACTTGGGTTTAATTCATTTAGCAA
TAAAAGCGTAGAAGCCTTTTCTTTCCCCATGCAATAAAGATTGGAGCTCATTTTTCATCCAGAATGAGTGAGTTCTTTTT
GCACTGGTGCTTGGTCTTTTGCTCGATATATACATATAAGTATGAATGTGTGTGTTTGTATAGATGTGTATGTAATTATG
AGATAGCATGTAAGCTGGACCCAACTGGTTTGAATGGAGATTTATGAATGTGTATGCATGAATCCGTATATGGATTTATG
TAAGTTTAATCCATATTTGCTTGTGTAAAAATATTTCCTTCTACGAGTGTGACTCTCTTCTCTTGGTTCAATAGAAGTTT
ATTGCTCCAAACTTCCCCCTAGCCTGACAAGCAAGAGGCATCCAGACCAGAGGGGAAAGACTCTAGCAATTAATCCTTTA
CTTAATTCCCCAATCCTCCTTTTCTTAAAGAATTTTCATAGGAAACTTTTTAGAGAGAACATAGAAATAAAGGATGAAAT
CACGTTTCAATGTGTAGCAGGCAGGGAGTTAGAGCTGAGCAGTTCCTGTGGAGGTAGAACAAAGGCTACTTTACTTAAAT
CCCCTGGTGTTTTATGATGAGGTAGGTGCTGAATACCAGAGACTGCAGCTTCCGGCCTCAGTGGAACTCAGGCAGGTCTG
CTACTGACACAAAGTGACTTAGACTTAAGGTAGGTGTTATAAAGCAACCCTAAATATCTGTAAGATCAAGTCTTACTCCT
GTCGATGCTCCCTCGGCTGCCTCATGAAGAACCAAGAAGAACGAAGAACAGCCAGGGCTGCCCCCGTTCCCATCCCACCC
CAGATTAGGTTCGATGGAGTGGGAAGACCTTAACTTTGAGCAGCACCACTCCATGAGCCAGGGACCGGAAGGCATCAAAA
AGGAACCGCGAGCACCAGCATTCACCTCTCTCTGCCTCCTGGCTGTAGACAGGGCGTGGTCAGCCGCCTCCCGCCCCTGC
TGCCTCAGATGGACTGGACCTGCCCACACTGCGAGCAAAATAAACCCTTCCTTCCTCAAGTTGCTTCTGCCAGGCATTTT
GTTACAGCAAGAAAATTAGATAAAAACCAGACTAAGTCCAGAAGGCCAGTGGGGGTTAATGCTTCCACACCAACTTGAAT
ATGAGGGTGGCTATAGCTACAGCTCATTTCAAAGGGTGTAGGTGGGCGTGAAGGTGCAAGTCTGTCATCCAGCACTCGGG
AAATGGAGGCAGGAGGACCAGGAGTTCAAGATCATCCCTGACTACATACGGAATAAGAAACCAGCCTGGACTGTACAAGA
TTCTGTATCAAATAATAGTAATACCACCGTCACTCCCTGCCTGCCATAAAGCTTCAGCCACATTTGAAACCTGACTGGTA
TCGGAGATGCACCGAGAAGTGGTCCGACTCGGTTTTGATTCCCTGGTGCTCCCTAACCTCCAATTTATTGACTTTGCTCC
TGAACTTCAGTGAGAAGAGGATTGTGGTGAAAGAGAAGCTGTGTAGAGAAGAGAAACTGCCCTGGGTTTGGGATCCCTGA
CTGCTAATGGTGATATCAGAATATTGGGGTATCTTTGAGAATGGCACAGATCTTGATAAGCTTGGATGGGGACAGGAGGG
GCCAGAGTCACAGAAAACTCAATTCACATAGTGTTTTTCTGAACCAAATTATGAATGAAACTAATGCCCAATTGAGGCAA
ACCTCCGAAGCCATAGTAGGACGATATGGATGCCGTTCAGGTTGTACAGTGAATGAGCTCTGCTGCATTAGCAGGTGACA
GCTTTTGGTGAGAGTTAATGTCCCTGCTGCTGCTGGAGTTAAATTTCCTTCTCATACTGAGCTTGCCTGGATTTCCTGTT
GGAAAAGCTCCAAGAGCAAGGCCATTAGCAGCCACTTACAAGGAGCTCCCGGATCCAAGCAGCGTGGCTGATGACACTGA
GACACTGATGAGCACTCTTGACCTTTCTGTAAGGAAGGACCGCTCACCAGGGAGTCAGGAAAAAAGCTGGTTGCAACAGA
GAAAACAGTAGTCCTTCACCTACTGGTAAAACTGTCACCCAACACACCGGCTCGTTCTGGATGGTTATTTTCTTCCTGGT
GCCAACTCCTGTAGGAAGTTTTAAACTTGCAAAGCCTATCTTGTAGCATAACAGAGCATACTGGCCCAAGGTGGGGTGCC
```

TTAGGTAAAATTATATGAGGAGTGATCATTCTAAGAACGGGTATATCTATAGAGACAGGGTAAATACTGTATTGGGTAAT
mK5F GGCATGCACTGTTCTATTGAACTTTTTTAGTAAAGGCGCTTGCTGTGTCTGCTGGAGATAACCTAGTACTAACCTTACCG
GACAGAGAAGCAGAACTGAAGGCCTCTGTTGAACCAGCGCCCCCTGCAGGTGAGAGTCTGGTGTTCTTATGTGACTCCGA Exon 5
mKi5F1 GACTGGTAGAGCTCAGGCATTCTCTGAGCCACTACCCGGCTATCAAAAGGCTTCAGACAACTTTAGTTCAAGAGATGCTT
AGGTGACATTTTAGACAAACCCCCTATAAAAATCAAGTGCGCTGAAACCTATTTGCTTTTCTTTTTTTCTTTTGGTTTTT
CGAGACAGGCTTTCTCTGTGTAGCCCTGACTGTCCTGGAACTCACTTTGTAGACCAGGCTGGCCTCGAACTCACACATCC 5’ arm
GCCTGTCTCTGCCTCCCAAGTGCTGGGATTACAGGCGTGCGCCACCACCGCCCAGCTTCTGCTTTTCATCAAAAGGAGAA
AAGATACAGTGAATTACACAGTTTAAGTGT

Trapping cassette

SA-F1 AAGGCGCATAACGATACCACGATATCAACAAGTTTGTACAAAAAAGCAGGCTGGCGCCGGAACCGAAGTTCCTATTCCGA
AGTTCCTATTCTCTAGAAAGTATAGGAACTTCGAACCCTTTCCCACACCACCCTCCACACTTGCCCCAAACACTGCCAAC FRT
TATGTAGGAGGAAGGGGTTG**GGACTAACAGAAGAACCCGTTGTG**GGGAAGCTGTTGGGAGGGTCACTTTATGTTCTTGCC **LAR3**
CAAGGTCAGTTGGGTGGCCTGCTTCTGATGAGGTGGTCCCAAGGTCTGGGGTAGAAGGTGAGAGGGACAGGCCACCAAGG
TCAGCCCCCCCCCCCTATCCCATAGGAGCCAGGTCCCTCTCCTGGACAGGAAGACTGAAGGGGAGATGCCAGAGACTCAG
TGAAGCCTGGGGTACCCTATTGGAGTCCTTCAAGGAAACAAACTTGGCCTCACCAGGCCTCAGCCTTGGCTCCTCCTGGG
AACTCTACTGCCCTTGGGATCCCCTTGTAGTTGTGGGTTACATAGGAAGGGGGACGGGATTCCCCTTGACTGGCTAGCCT
ACTCTTTTCTTCAGTCTTCTCCATCTCCTCTCACCTGTCTCTCGACCCTTTCCCTAGGATAGACTTGGAAAAAGATAAGG En2
GGAGAAAACAAATGCAAACGAGGCCAGAAAGATTTTGGCTGGGCATTCCTTCCGCTAGCTTTTATTGGGATCCCCTAGTT ‘intron_1/
TGTGATAGGCCTTTTAGCTACATCTGCCAATCCATCTCATTTTCACACACACACACCACTTTCCTTCTGGTCAGTGGG exon_2’
CACATGTCCAGCCTCAAGTTTATATCACCACCCCCAATGCCCAACACTTGTATGGCCTTGGGCGGGTCATCCCCCCCCCC
ACCCCCAGTATCTGCAACCTCAAGCTAGCTTGGGTGCGTTGGTTGTGGATAAGTAGCTAGACTCCAGCAACCAGTAACCT
CTGCCCTTTCTCCTCCATGACAACCAGGTCCCAGGTCCCGAAAACCAAAGAAGAAGAACCCTAACAAAGAGGACAAGCGG
CCTCGCACAGCCTTCACTGCTGAGCAGCTCCAGAGGCTCAAGGCTGAGTTTCAGACCAACAGGTACCTGACAGAGCAGCG
GCGCCAGAGTCTGGCACAGGAGCTCGGTACCCGGAAGATCTGGACTCTAGAGAATTCCGCCCCTCTCCCTCCCCCCCCCC
TAACGTTACTGGCCGAAGCCGCTTGGAATAAGGCCGGTGTGCGTTTGTCTATATGTTATTTTCCACCATATTGCCGTCTT
TTGGCAATGTGAGGGCCCGGAAACCTGGCCCTGTCTTCTTGACGAGCATTCCTAGGGGTCTTTCCCCTCTCGCCAAAGGA
ATGCAAGGTCTGTTGAATGTCGTGAAGGAAGCAGTTCCTCTGGAAGCTTCTTGAAGACAAACAACGTCTGTAGCGACCCT
TTGCAGGCAGCGGAACCCCCCACCTGGCGACAGGTGCCTCTGCGGCCAAAAGCCACGTGTATAAGATACACCTGCAAAGG
CGGCACAACCCCAGTGCCACGTTGTGAGTTGGATAGTTGTGGAAAGAGTCAAATGGCTCTCCTCAAGCGTATTCAACAAG
GGGCTGAAGGATGCCCAGAAGGTACCCCATTGTATGGGATCTGATCTGGGGCCTCGGTGCACATGCTTTACATGTGTTTA IRES
GTCGAGGTTAAAAAAaCGTCTAGGCCCCCCGAACCACGGGGACGTGGTTTTCCTTTGAAAAACACGATGATAAGCTTGCC
ACAACCATGGAAGATCCCGTCGTTTTACAACGTCGTGACTGGGAAAACCCTGGCGTTACCCAACTTAATCGCCTTGCAGC
lacZ-F3 ACATCCCCCTTTCGCCAGCTGGCGTAATAGCGAAGAGGCCCGCACCGATCGCCCTTCCCAACAGTTGCGCAGCCTGAATG
GCGAATGGCGCTTTGCCTGGTTTCCGGCACCAGAAGCGGTGCCGGAAAGCTGGCTGGAGTGCGATCTTCCTGAGGCCGAT
ACTGTCGTCGTCCCCTCAAACTGGCAGATGCACGGTTACGATGCGCCCATCTACACCAACGTGACCTATCCCATTACGGT *lacZ*
CAATCCGCCGTTTGTTCCCACGGAGAATCCGACGGGTTGTTACTCGCTCACATTTAATGTTGATGAAAGCTGGCTACAGG
AAGGCCAGACGCGAATTATTTTTGATGGCGTTAACTCGGCGTTTCATCTGTGGTGCAACGGGCGCTGGGTCGGTTACGGC
CAGGACAGTCGTTTGCCGTCTGAATTTGACCTGAGCGCATTTTTACGCGCCGGAGAAAACCGCCTCGCGGTGATGGTGCT
GCGCTGGAGTGACGGCAGTTATCTGGAAGATCAGGATATGTGGCGGATGAGCGGCATTTTCCGTGACGTCTCGTTGCTGC
ATAAACCGACTACACAAATCAGCGATTTCCATGTTGCCACTCGCTTTAATGATGATTTCAGCCGCGCTGTACTGGAGGCT
GAAGTTCAGATGTGCGGCGAGTTGCGTGACTACCTACGGGTAACAGTTTCTTTATGGCAGGGTGAAACGCAGGTCGCCAG
CGGCACCGCGCCTTTCGGCGGTGAAATTATCGATGAGCGTGGTGGTTATGCCGATCGCGTCACACTACGTCTGAACGTCG
AAAACCCGAAACTGTGGAGCGCCGAAATCCCGAATCT**CTATCGTGCGGTGGTTGAAC**TGCACACCGCCGACGGCACGCTG **lacZ-R3**
ATTGAAGCAGAAGCCTGCGATGTCGGTTTCCGCGAGGTGCGGATTGAAAATGGTCTGCTGCTGCTGAACGGCAAGCCGTT
GCTGATTCGAGGCGTTAACCGTCACGAGCATCATCCTCTGCATGGTCAGGTCATGGATGAGCAGACGATGGTGCAGGATA
TCCTGCTGATGAAGCAGAACAACTTTAACGCCGTGCGCTGTTCGCATTATCCGAACCATCCGCTGTGGTACACGCTGTGC
GACCGCTACGGCCTGTATGTGGTGGATGAAGCCAATATTGAAACCCACGGCATGGTGCCAATGAATCGTCTGACCGATGA
TCCGCGCTGGCTACCGGCGATGAGCGAACGCGTAACGCGAATGGTGCAGCGCGATCGTAATCACCCGAGTGTGATCATCT
lacZ-F8 GGTCGCTGGGGAATGAATCAGGCCACGGCGCTAATCACGACGCGCTGTATCGCTGGATCAAATCTGTCGATCCTTCCCGC
CCGGTGCAGTATGAAGGCGGCGGAGCCGACACCACGGCCACCGATATTATTTGCCCGATGTACGCGCGCGTGGATGAAGA
CCAGCCCTTCCCGGCTGTGCCGAAATGGTCCATCAAAAAATGGCTTTCGCTACCTGGAGAGACGCGCCCGCTGATCCTTT
GCGAATACGCCCACGCGATGGGTAACAGTCTTGGCGGTTTCGCTAAATACTGGCAGGCGTTTCGTCAGTATCCCCGTTTA
CAGGGCGGCTTCGTCTGGGACTGGGTGGATCAGTCGCTGATTAAATATGATGAAAACGGCAACCCGTGGTCGGCTTACGG
CGGTGATTTTGGCGATACGCCGAACGATCGCCAGTTCTGTATGAACGGTCTGGTCTTTGCCGACCGCACGCCGCATCCAG
CGCTGACGGAAGCAAAACACCAGCAGCAGTTTTTCCAGTTCCGTTTATCCGGGCAAACCATCGAAGTGACCAGCGAATAC
CTGTTCCGTCATAGCGATAACGAGCTCCTGCACTGGATGGTGGCGCTGGATGGTAAGCCGCTGGCAAGCG**GTGAAGTGCC** **lacZ-R5**
TCTGGATGTCGCTCCACAAGGTAAACAGTTGATTGAACTGCCTGAACTACCGCAGCCGGAGAGCGCCGGGCAACTCTGGC
TCACAGTACGCGTAGTGCAACCGAACGCGACCGCATGGTCAGAAGCCGGGCACATCAGCGCCTGGCAGCAGTGGCGTCTG
GCGGAAAACCTCAGTGTGACGCTCCCCGCCGCGTCCCACGCCATCCCGCATCTGACCACCAGCGAAATGGATTTTTGCAT
CGAGCTGGGTAATAAGCGTTGGCAATTTAACCGCCAGTCAGGCTTTCTTTCACAGATGTGGATTGGCGATAAAAAACAAC
TGCTGACGCCGCTGCGCGATCAGTTCACCCGTGCACCGCTGGATAACGACATTGGCGTAAGTGAAGCGACCCGCATTGAC
CCTAACGCCTGGGTCGAACGCTGGAAGGCGGCGGGCCATTACCAGGCCGAAGCAGCGTTGTTGCAGTGCACGGCAGATAC
ACTTGCTGATGCGGTGCTGATTACGACCGCTCACGCGTGGCAGCATCAGGGGAAAACCTTATTTATCAGCCGGAAAACCT
lacZ-F1 ACCGGATTGATGGTAGTGGTCAAATGGCGATTACCGTTGATGTTGAAGTGGCGAGCGATACACCGCATCCGGCGCGGATT
GGCCTGAACTGCCAGCTGGCGCAGGTAGCAGAGCGGGTAAACTGGCTCGGATTAGGGCCGCAAGAAAACTATCCCGACCG
CCTTACTGCCGCCTGTTTTGACCGCTGGGATCTGCCATTGTCAGACATGTATACCCCGTACGTCTTCCCGAGCGAAAACG

GTCTGCGCTGCGGGACGCGCGAATTGAATTATGGCCCACACCAGTGGCGCGGCGACTTCCAGTTCAACATCAGCCGCTAC
AGTCAACAGCAACTGATGGAAACCAGCCATCGCCATCTGCTGCACGCGGAAGAAGGCACATGGCTGAATATCGACGGTTT
lacZ-F2 CCATATGGG**GATTGGTGGCGACGACTCCT**GGAGCCCGTCAGTATCGGCGGAATTCCAGCTGAGCGCCGGTCGCTACCATT **lacZ-R7**
ACCAGTTGGTCTGGTGTCAAAAATAATAATAACCGGGCAGGGGGGATCTAAGCTCTAGATAAGTAATGATCATAATCAGC
CATATCACATCTGTAGAGGTTTTACTTGCTTTAAAAAACCTCCCACACCTCCCCCTGAACCTGAAACATAAAATGAATGC SV40 pA
AATTGTTGTTGTTAACTTGTTTATTGCAGCTTATAATGGTTACAAATAAAGCAATAGCATCACAAATTTCACAAATAAAG
CATTTTTTTCACTGCATTCTAGTTGTGGTTTGTCCAAACTCATCAATGTATCTTATCATGTCTGGATCCGGAATAACTTC loxP
GTATAGCATACATTATACGAAGTTATGTTTAAACGGCGCGCCCCGGAATTCGCCTTCTGCAGGAGCGTACAGAACCCAGG
GCCCTGGCACCCGTGCAGACCCTGGCCCACCCCACCTGGGCGCTCAGTGCCCAAGAGATGTCCACACCTAGGATGTCCCG
CGGTGGGTGGGGGGCCCGAGAGACGGGCAGGCCGGGGGCAGGCCTGGCCATGCGGGGCCGAACCGGGCACTGCCCAGCGT
GGGgCGCGGGGGCCACGGCGCGCGCCCCCAGCCCCCGGGCCCAGCACCCCAAGGCGGCCAACGCCAAAACTCTCCCTCCT Beta actin promoter
CCTCTTCCTCAATCTCGCTCTCGCTCTTTTTTTTTTTTCGCAAAAGGAGGGGAGAGGGGGTAAAAAAATGCTGCACTGTGC
GGCGAAGCCGGTGAGTGAGCGGCGCGGGGCCAATCAGCGTGCGCCGTTCCGAAAGTTGCCTTTTATGGCTCGAGCGGCCG
CGGCGGCGCCCTATAAAACCCAGCGGCGCGACGCGCCACCACCGCCGAGACCGCGTCCGCCCCGCGAGCACAGAGCCTCG
CCTTTGCCGATCCTCTAGAGTCGAGATCCGCCGCCACCATGATTGAACAAGATGGATTGCACGCAGGTTCTCCGGCCGCT
TGGGTGGAGAGGCTATTCGGCTATGACTGGGCACAACAGACAATCGGCTGCTCTGATGCCGCCGTGTTCCGGCTGTCAGC
GCAGGGGCGCCCGGTTCTTTTTGTCAAGACCGACCTGTCCGGTGCCCTGAATGAACTGCAGGACGAGGCAGCGCGGCTAT
CGTGGCTGGCCACGACGGGCGTTCCTTGCGCAGCTGTGCTCGACGTTGTCACTGAAGCGGGAAGGGACTGGCTGCTATTG *Neo*
GGCGAAGTGCCGGGGCAGGATCTCCTGTCATCTCACCTTGCTCCTGCCGAGAAAGTATCCATCATGGCTGATGCAATGCG
GCGGCTGCATACGCTTGATCCGGCTACCTGCCCATTCGACCACCAAGCGAAACATCGCATCGAGCGAGCACGTACTCGGA
TGGAAGCCGGTCTTGTCGATCAGGATGATCTGGACGAAGAGCATCAGGGGCTCGCGCCAGCCGAACTGTTCGCCAGGCTC
AAGGCGCGCATGCCCGACGGCGAGGATCTCGTCGTGACCCATGGCGATGCCTGCTTGCCGAATATCATGGTGGAAAATGG
CCGCTTTTCTGGATTCATCGACTGTGGCCGGCTGGGTGTGGCGGACCGCTATCAGGACATAGCGTTGGCTACCCGTGATA
TTGCTGAAGAGCTTGGCGGCGAATGGGCTGACCGCTTCCTCGTGCTTTACGGTATCGCCGCTCCCGATTCGCAGCGCATC
GCCTTCTATCGCCTTCTTGACGAGTTCTTCTGAGCGGGACTCTGGGGTTCGAAATGACCGACCAAGCGACGCCCAACCTG
CCATCACGAGATTTCGATTCCACCGCCGCCTTCTATGAAAGGTTGGGCTTCGGAATCGTTTTCCGGGACGCCGGCTGGAT
GATCCTCCAGCGCGGGGATCTCATGCTGGAGTTCTTCGCCCACCCCCCGGATCTAAGCTCTAGATAAGTAATGATCATAA SV40 pA
TCAGCCATATCACATCTGTAGAGGTTTTACTTGCTTTAAAAAACCTCCCACACCTCCCCCTGAACCTGAAACATAAAATG
AATGCAATTGTTGTTGTTAACTTGTTTATTGCAGCTTATAATGGTTACAAATAAAGCAATAGCATCACAAATTTCACAAA
TAAAGCATTTTTTTCACTGCATTCTAGTTGTGGTTTGTCCAAACTCATCAATGTATCTTATCATGTCTGGATCCGGGGGT
PNF ACCGCGTCGAGAAGTTCCTATTCCGAAGTTCCTATTCTCTAGAAAGTATAGGAACTTCGTCGAGATAACTTCGTATAGCA FRT
TACATTATACGAAGTTATGTCGAGATATCTAGACCCAGCTTTCTTGTACAAAGTGGTTGATATCTCTATAGTCGCAGTAG loxP
R2R GCGG

mKi5F3 ATTCGGACCTAGTGAGGTTGAAGACACTTCAGTCTACTATATAGTGAGCTCCAGGCCAGCCAGAGCTCACAGTGAGAGCC Critical region
TGTCTCAAAACAACAACAAGAAGGAATTATATGGATAATCAGGTGTTGGCAAGAGCCCAGAAGGAAGGTTTTCAAATGCT
AATAGTAAAGTTGGGGAGGTTTAATTTTTAGTCCCCATTTTCTATCATTTGTGTACTTTCATTATCAGTTTGTTTTTGTG
TTTGTTTGTTTCTCTTCATTTGCAAA**GACACAGAGACCGGAGATTC**CTCTCTGGGACGAGCCTTGGAAGCAGAGCTCTGT **mKi5R3**
GTTTCTAAGGCAGTTGATGCTGTTGGTTTAAAGCATTCATCTCCTTAGGGTTATAAGGTGACCTCTCAGATCTGATAAGC
ATTCGTGTTTTCCCTCAGATACAACTTACTCCTATGAATGGAGTTTAATGAGCCACCCAGTAGACTTCCAAGGTAAAATC Exon 6
AAACAAGAAAACAAGCCGACTCTTCACCTCTCTCAAGTAAGTAACAAGGCAGGTTGCACAGCTCAGACTCCAGTGAGCAA
AGTCTTGGCTGTTGAATGCAGAATTCCGTTTTAGGATTAAGAGTGGATAAAGGAGCCCGGCAT**GATGGATGGCATGTGCC** **mKi6R1**
TGCAGTGTTATCTACTTAAAAGTGAGGgCAAAAAGATTGCTTCGTCCTAGAAGTT

loxP cassette

GAGATGGCGCAACGCAATTAATGATAACTTCGTATAGCATACATTATACGAAGT**TAT****GGTCTGAGCTCGCCATC**AGTTCA loxP **loxP3R**

TCAGTAGCCTTCCTTCATTCGAATTGCTTGAAATAACAGCGCTGAAGAAAGAGGCTGGAGGTCGGGGCCATAATGTGTGG 3’ arm
TAGGTTCAAAGCCTGGGTCTAATTCTTTCAGTCTTTACAGTGTTAAAGATATCTCAGGTGTCCACGAGGTATGTTCCGTT
TTAGTTCACAAGGTGCTTAGATTGTCAAATACTGCTCCACACAAGAAGACA**CATACCACTTCCTTGGCTCC**AAAAGGAAG **mKi6R3**
CTCGGGGTCTGGCTTCTTATGAGGTGACTGTCTTCCTGTTCAAGGCTGTACCAATCCCAGTGA**CTTACCAGCTCACGTCA** **mKi6R4**
TTGCTGCGTGACTGAGCTATTTCTCCAAGTGTGGTACCCAGACCCCTGCCAGTAACACGTGTTAGAAATCTCTGACTCGA
GACCCTGTTGCTGGTCAGTGTAGAGTCCCTGCTGAGCATATGTGAGGCCCTGGGCTCACTTGCAGCACTGACTCACCCCC
ACTCCACAAAAACCCAAGTCCAGCATGATTCCATACATGCCCATCTGAGGGCTGCTGTCCTTGGCTGATTTGAAGTCTGT
GACATCTGTTACTAAGCAAAAGGCCTCTGTAGGTAACTTAGTATCTTAGAGTGCAGCTCTCAAATGTGTAGCAGTCCTAG
ACAGACTAAAATGAAGCTTTCTCCCAAGTCTTTGGGGGCATTGTAGGCTTTTCTCGGAGGCAGATGCCCCTGTCTTCAGA
TGCTCACTTGTGTATCTGTTTCCATCTTGCAGTTATCTGTGGGACTCTATGCCTTCAGAGTGGCTGTTTCTAGTGAGAAT Exon 7
GCATTTGGAGAAGGCTATGTCAATGTCACGGTTATGCCAGGTGAGTCTGTTGGGAGAATGGCATCTTGGCTGTGCTAGTC
TCAAGTTCTGGCACCTGGATGACATGGAGGTATAGATTGATGACATGACTGACCCATGGAATGGTGAAGGGGAAAGACTT
TACTGTAGTAGATACTGAGGGAGCCTAGAAGCCAGCATGGGCTTTGATATGCTGGTAGGGACCACAAGAAACCATATGTC
CCTTTGGCCAGAGGTAAAGGTGCCTTTTGGTAGAGGGGAACTGGCTTCCCAAGTTCCTGAGGAATGCTGGCTTCTACCTA
ACCACCAGAAATCTTATAGTTAAGGTTGAGCTCATTTCTGGATATTTGGACTGCCTTTTGGAGTTTGGACCAGACAGTGT
TTTAATACAAACGCTTTTATTTTTTATTTATACAAGTTAGACTTGTGTAGTCGTGAAGAGACACCGTGACCAACTCTTAT
AAGGCAACTCTTATAAAGGAAAGCAGTTAATTAAGGGCTCGCTTTCCGATTCAGAGGCTTAGGTCATTATCACTGTGGCA
GGGAGCATGGCAGCAGCGGGCAGGCAGGCAGGCAGGCAGGCACGGTGTGGTGCTGGAGGAATGGCTGAAAGCTCTGCATC
CTGACCCACAGGCAGCAGGTAGGCAGGCAGGCAAGGTGGCAGGTGGGGAGAGGGAAGAGAGGTGGGCCAGGTGTAGGCAT
TTGAAACCTCAGTGCCTACCCCCCAGTGACACAGTGGCTTCCTCCAGCAAGGCCACACTTCCTAATCCTTCTCAACAGCC

CCACTCTCTGGTGACGAAGCATTCAAATATACGAGCCTTTGGAAGCTGTTCTCGTTAAACAACCACAGGCACCATTGTAG
AGAAAATGTACAAGGATTAGGTTTGGTCTTTTTGTTTCCTTTTGCTTTTTTTTTTTTTTTTTTTTAGCTCTAGTATTCTCT
TGACATGGCCACTCAAGTATACCAGGAAGCTGCTTGCTCTTCTTGAATGATAACAAACTGTACCTTGGCCCTCCCTGTCT
AACACTTACTCTGTCCACTTTCTTGCCTCATGATTTAGCTTTCATATAGGAGTATGGCTATTTCAAAAATGCATTCATCT
GTGCACCTAAAGAATCAGGGGCACATGTATGTCATGGGCTGATTAGCAAGCCCATCCCTGGGTCATAATTCAGTGTATTT
GTGTGGATTGATTAGAGCAGGTGACCTTCAGTCCCGGTGGGTACACTCTGCTCTATCTATGTCCTGGCTCCCGGTTTCCA
CTTACCCACCAGCTCAGCCGAGCCATTAGCTTACATCAGCATGTTAGCAGAAGCTCCACCTCCTACCCATCTCTCTGGTG
GGCCGAAGCTTTTCCATAGTCTACATAATACAGCTGAAGTCTGGTGATGATGGAAGTACCTTCAATCAAAGATTTAAGAT
GCTATTCATGGTCTGAGAATGTAGCTCAGTTAGCAGAGTTCTTGCCCAGCACTGAATACACTTGTATTCACCCTGTGATT
CTAGCACTTGGGAGGCCCAGACAGGGGGATCAGGACTTTAAAAGGTTATGCATAGTGAATTGGAGGCTACGCTCATGTCT
TAGTTGGGGTTTCCATTGTTGTGAAGAGACACCATAACCAAGGGACTCTTCATTGGTGATTTAATCTGAAAATTGCAGTT
TCAGAGGTTTAGTTCGTTATCATCATGGTGAGAAGCATGGCAGCTTGTAGGCAGACATGGTGAGGGAGGAGGCGAGAGTT
CTACATCTTGATCTGCAGGCGTCAGGGAGAGACTGTGTTCTGCAGGCAGCCAGAGGAGGCTCTTATTCCACACTGGGCAG
AGCTTGAACATAGGATATCTCAAAGCCTGCCTACACAGTGATATACTTCCTCCAACAAGGCCACACCTCCTAAGAGTGCC
ACTCCCTATGGCCCAGCATTGAAACACATGAGTCTCTGGGGGTCAAAGCTATTCAGATCACTACTCCTATCATAAAGCCC
TGCAATCTAGCCTTGCTTTTCTGTAACCACAGTACACATTGTATGACTTACTCCTTGAGTCTGACTTTACCTATAGAATT
GTAGACTGACAGACAAGCTCAAAATCCACAGGAGGGGTTTGACAGTTTGGGGAGTTTCCCACAGTTGAGGCCCAGGGAGG
TGTGTGTGGGAGGAGAGGAGCAGCGGGTATGTGTGTGAAGACCTCTACAAGATGTTTAGAATTAGCATTTAGAAAGAGGG
TGCCTTTGTGTGTGTCTGACTGGTGTGTCCGGTGTAGGCTAAGTTGCTGTCTTTTTTTTGTCTCACAGCTGCAAGAGTCA
ACCAGCCACCTGTAGCTGTTGTTTCTCCCCAGACACAAGAGCTCAGTGTGCCTTTGACCTCAGCCCTGATCGATGGCAGC Exon 8
CGTGCGTATCCTCCATGTTCGGTGGTTTCTGTTTGTTTATACTGTCAGATTGAGTGCTTTGTGTTAGGGCCAAGAAGGCT
TACAGTGACACCATGATAAACTGGGGGTTCTTTTAGCTTCCTGTCCTAGGCTCCTCAGCTCCCAGTTGAGAGTGGTAGCA
TGCCTGTAACCTTCCTGTCACCCTAGCATTTGGGTGCCAAAGGCAGGAGGGTCAGGAATTCAAGGTCATCTTTAGTGACT
TAGGGAGTTCCAGGCCAGCCTGGGATACATGAAACATTGTCTAAAACAAACTACAAACAAACAAATACAGCATTCATTAA
GGTCAGATATTTGATTTGTAATTCAAATAATTACTAAGGATTCTTTTGTGAATCGTGTAGCAGTTGTAAGATTAGCAATT
GGACAGAGCCTTTCAGTCCGAATGAGGTTGGCTTCAGTGATAGTCAGTAGCATTTCACTTTACTAATTTAGTCAGAGTTT
ATTCTTTAAGTGGAGGATAAGGATGGTGTAACTTTGGGTGTCCAGACAAATGAGAATCCAGTGTTTGGCTTGGTTTAACA
GTGTAGAGTGCTCTGCAACTGTTTTTACATTCATCCAGTTACAAGGTTAACCCCACGGCCGGCTGAGAGGTAAAAGGTGT
GGTCATACCGGTTGCTCCCGAGGGTTGTTCTGTCTACTAGTCTGCGGATGTGGACGCGACAGCAATGTGACTGCTCGAAT
CTATTCGGTGTCATGTCTACATCCTCCCCCTTCTCCAGTGTGAACATACAGTACAGTTGCTCGTTTATTTGCTTGCTTGC
TTTCTCATACAGCTCAGGCTCTTACCACACTTGCTATGTATTGAAGGTTGACCTTCACCTCCTGATGACCTTTTGACCCT
TTCCCAAGTTCTGGGATTCTAGGCACCTGTATCCCCATGCCTGGCTGACAAAGGAGATGCTTGTATGAGAAAAGGCCTCA
CAGTTTTAAAAAAAATCTTTTGATTCTGACTAGCTTAGTTACAAAAATCATACAGGATTATTAGTTGATCATAATAAATA
AGCACATAAATTTTTTCCCTAAATGAATTTGATGAAAAGCCAGCTTCCTTCTAGATCTATGGGTCTACATGGCACATTGT
TGCTCTCTTTTATTATTTGTTTGTTTGTTTTGTGATATGGAGACTGAACCCACCACGCCTTTGTAGATGTAGCATGTAAT
TATAAAAAAACAATCCACGCTGTGGGCGGCTAGGCACCAGTGGTCTCTGTCTGTTCCCAGCCCAGTACATAGCCTGAGAT
CGAGACCGTGTGTTTTTCCTAGCCAACACCTCTCCCACAAGTGTCAGCTCTTTCTCTCTAGAGCTCCGAATTCCCTCTCT
TGATCCCAGAGGCCAGCTGCAGTTACCTCTCCCCTGGAGCTTGGTCCTTGCCCAGTCTTCCCGCTCCAAAGCCTGGCCAA
ATTCTGCCCCAGCTAAATTCTTTCCCTCTGGCCCACCTGAGTTGTGGTGAATGGAATACACCAGATGATCTTAGTTTAGA
CTCAACAGATAACACAACCACTGCGGGTGGACTCTATTGTCCTAAACTCATCAACTATTCTAAGTTGGAAATATTCCAGT
GGCAGAATCCGCCACCAGATCTAACCATTCCCAGTCTACATCCTGGTCCTCTTGCTTCCACCTTGCCAGACAAGAAAGGC
TGTTCCTCCCTCTGCGTCCCTCTTCCTCTCTCAGAACGGAAGGCCAATCTCCTCCTTTTCACCCAGCCATTGGCTTCTTG
TTGTCTTTATTATCCAATCAGGGGAAAGTTCTGTTACAGTGGATGCTAGGCAAACACTTCTGTCACTTAGCTGTATCCCC
AGCCCTGTTCTTTTTGTGATATAAGGTCTCACCTTGTAGCCTAGTTTGGCCTTGAACTCATGGAGATAATCCTGCCTTAG
CTTCCTGAATGCCATTGAACTACCATGCCAAGC**CACAGCCTCTTTCTCCACTACTGG**GGATCAAACTCAGGGCTT**CACAT** GR2
GCATGGAAGTTAAGCACTGTACCACTGAGCTACAGTCCTAGCCTTCTTCTCTTTTCATGTGTGTGTGTGTGTGTGTGTGT GR4
GTGTGTGTGTGTGTGTGTGTAGTGTGCACATCATGCCATACATGTAAAGTTCTGAGGACACCTTTTTTTTTTCTTTCTTT
TTTATTAGATATTTTCTTTATATACATTTTAAATCCCGAAAGTTACCTATACCCCGCCCCTGCCCTGCTCTCCTACCCAC
CCACTCCTACTTGTTGGCCCTGGCGTTCCCCTGTATTGGGGAATATAAAGTTTGCAATACCAAGGGGCCTCTCTTCCCAG
TGATGGCCGACTAGGCCATCTTCTGCTACATATGCAGCTAGAGACATGAGCTCTGGGGGTACTGGTTAGTTCATATTGTT
GTTCCTCCTATAGGGTTGTAAACCCCTTCAGCTCCTTGGGTGCTTTCTCTAGCTTCTCCATTAGGGGCCCTGTGTTCCAT
CTTATAGATGACTGTGAGCATCCACTTCTGTATTTGCCAGGCACTGGCATAGCCTCATACGAGACAGCTATACCAGGGTC
CCTTCAGCAAAATCTTCCTGGGATATGCAATAGTGTCTGGATTTGGTGGCTGATTATGGATGGATCCCTGGGTGGGGTA
GTCTCTGGATAGTCCATCCTTTCATCTTAGCTCCAAACTTTGTCTCTGTAACTCCTTTCATGGGTGTTTTGTTCCCTATT
CTAAGGAGGAATGAAGTATCCACACGTTGGTCTTCCCTCTTCTTGATTTTCTTGTGTTTTGCAAATCATATCTTGGGTAT
TCTAAGTTTCTGGGCTAATATCCACTTATCAGTGAGTGCATATCTAATGACTTCTTTTGTGATTGGGTTACCTCACTCAG
GATGATATCCTCTAGATACATCCACTTGTCCAAGAATTTTATAAATTCATTGTTTTTAATAGCTGAGTAGTACTCCATTG
TGTAGATGTACCACATTTTCTGTATCCATTCCTCTGTTGAGGGGCATCTGGGTTCTTTCCAGCTTCTGGCTATTATAAAT
AAGGCTGCTATGAACATAGTGGAGCATGTGTTCTTATTACCAGTTGGAACATCTTCTGGGTATATGCCTGGGAGAGTTAT
GGCTGGATCTTCCGGTAGTACTATGGAGGACAACTTTTGGTAGTCAGTTCTCTTCTTCCACCTTGTGGGTCCAAGCAACT
GAG**CTCTTCAACGTGACTTCAACGTGC** GR1

Fig. S4 PCR and genotyping details

Genotyping of mice		Primers		Expected band sizes				
				wt	tm1a	tm1b	tm1c	tm1d
PCR #	To detect:	Forward	Reverse	wt	KO1	Null-Z	Flx	Null
KO1-1	*Kiaa0319 locus*	GF1	LAR3		5834	5834		
KO1-2	*Kiaa0319 locus*	mKi5F1	lacZ-R7		5082	5082		
KO1-3	*Kiaa0319 locus*	lacZ-F1	mKi6R3		3760	1032		
KO1-4	*Kiaa0319 locus*	PNF	GR1		7375			
KW	*Kiaa0319 locus*	mKi5F3	mKi6R2	785				
KK	*Kiaa0319 locus*	PNF	mKi6R1		817			
KKF	*Kiaa0319 locus*	R2R	mKi6R1		663		663	
KKZ	*Kiaa0319 locus*	mK5F	LAR3		675	675		
KZ	*Kiaa0319 locus*	lacZ-F2	mKi6R3		3376	648		
KWF	*Kiaa0319 locus*	mK5F	mKi5R3	815	7879		975	
KN(F)	*Kiaa0319 locus*	SA-F1	mKi6R4		8219	5491	1315	498
4	***lacZ***	lacZ-F8	lacZ-R5		635	635		
4a	***lacZ***	lacZ-F3	lacZ-R3		871	871		

PCR #	To detect:	Forward	Reverse	wt	Flp	Sox2-cre
5	***Flp insertion locus***	oIMR1348	oIMR1349		725	
6a	***cre insertion locus***	Cre-F2	Cre-R6			820
6b	***cre insertion locus***	Cre-F3	Cre-R5			600
7	***cre insertion locus***	AGp-F2	Cre-R2			552

All	Internal control	oIMR7338	oIMR7339	324	ALL

	Sex	Primers		Expected band sizes	
PCR #	To detect:	Forward	Reverse	Female	Male
Sex a	*Sry locus*	mSry-F	mSry-R		402
Sex b	*Sry locus*	mSry-F1	mSry-R1		272

All	Internal control	mIL3_F	mIL3_R	544	544

Expected PCR results for different *Kiaa0319* allele combinations

	Kiaa0319 locus		PCR #							
Gen. #	Allele 1	Allele 2	KW	KK	KKF	KKZ	KZ	KWF	KN(F)	4/4a
1	wt	wt	+	–	–	–	–	+	–	–
3	KO1	wt	+	+	+	+	–	+	–	+
5	KO1	KO1	–	+	+	+	–	–	–	+
13	Null-Z	wt	+	–	–	+	+	+	–	+
15	Null-Z	Null-Z	–	–	–	+	+	–	–	+
7	Flx	wt	+	–	+	–	–	[+]+	[+]	–
9	Flx	Flx	–	–	+	–	–	[+]	[+]	–
17	Null	wt	+	–	–	–	–	+	+	–
19	Null	Null	–	–	–	–	–	–	+	–

Note: [+] indicates the bigger band of those that can be obtained from different alleles (pink-highlighted in PCR table)

PCR profile:

95ºC	15 min	
94ºC	30 sec	34 cycles
60ºC	30 sec	
72ºC	45 sec	
72ºC	10 min	
15ºC	infinite	

Ann. Temp. reduced to 58ºC for PCRs #5, #Sex-a and #Sex-b

Fig. S5 Normal lamination in early developmental stages

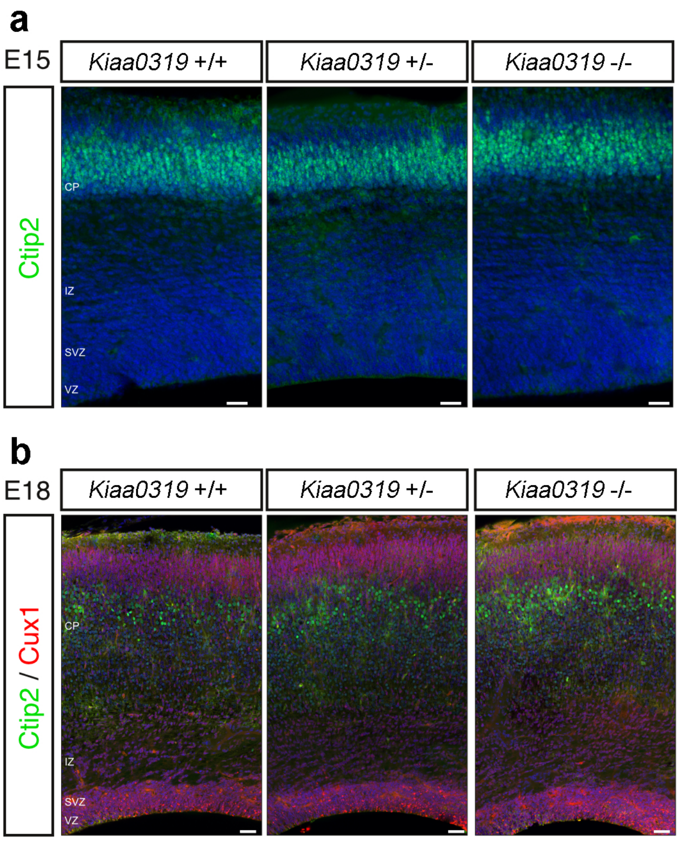

Fig. S6 Generation of *Null* allele by Cre expression in *Flx* electroporated cells

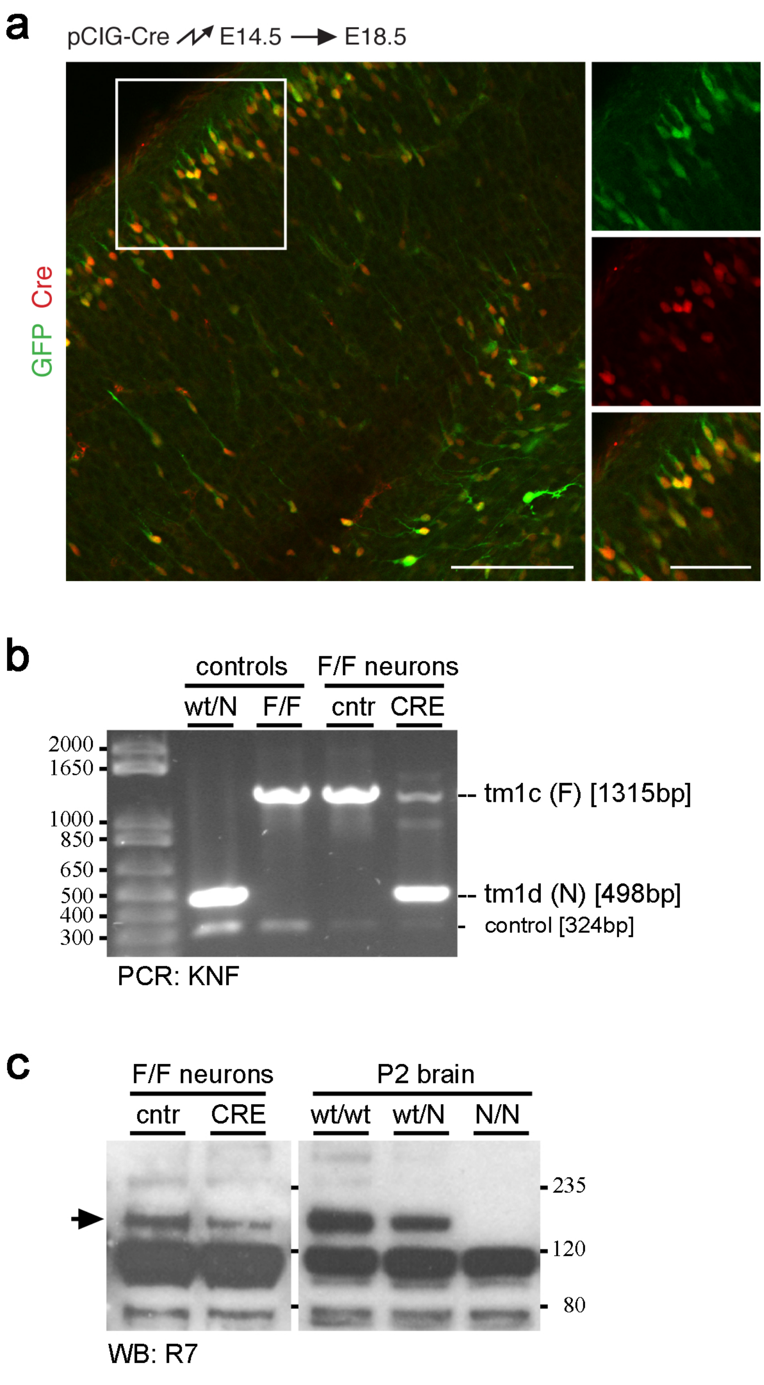

Fig. S7 Behavioural tests

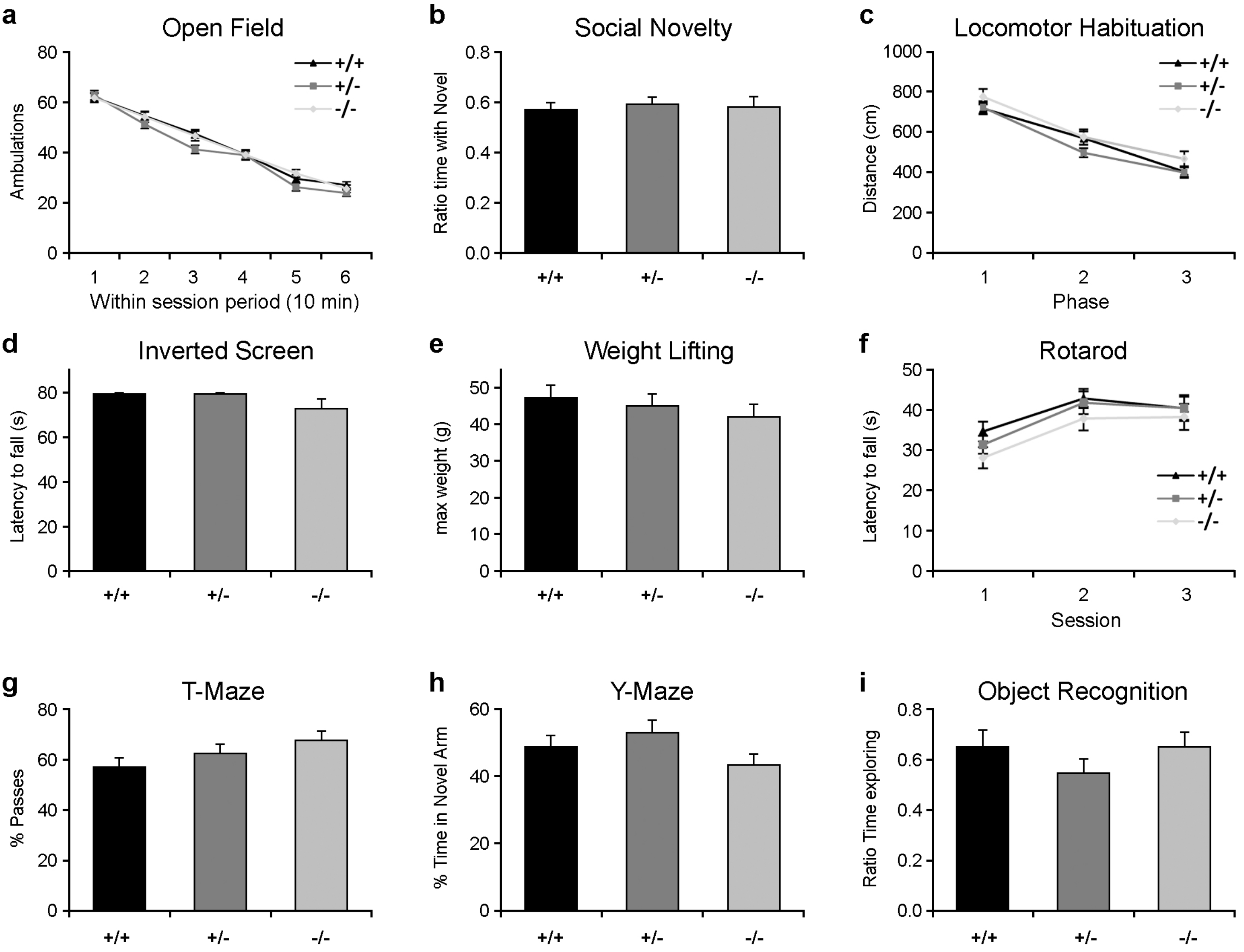

Supplement: Supplementary file 1 — Supplementary material 1 (PDF 12406 kb) [file 429_2016_1282_MOESM1_ESM.pdf]
